# Systemic 4-1BB stimulation augments extrafollicular memory B cell formation and recall responses during *Plasmodium* infection

**DOI:** 10.1016/j.celrep.2025.115528

**Published:** 2025-04-11

**Authors:** Carolina Calôba, Alexandria J. Sturtz, Taylor A. Lyons, Lijo John, Akshaya Ramachandran, Allen M. Minns, Anthony M. Cannon, Justin P. Whalley, Tania H. Watts, Mark H. Kaplan, Scott E. Lindner, Rahul Vijay

**Affiliations:** 1 Discipline of Microbiology and Immunology, Rosalind Franklin University of Medicine and Science, North Chicago, IL, USA; 2 School of Graduate and Postdoctoral Studies, Rosalind Franklin University of Medicine and Science, North Chicago, IL, USA; 3 Center for Cancer Biology, Immunology and Infection, Rosalind Franklin University of Medicine and Science, North Chicago, IL, USA; 4 Division of Biology and Biomedical Sciences, Washington University, St. Louis, Missouri, USA; 5 Deparment of Veterinary Biochemistry, Kerala Veterinary and Animal Sciences University, Thrissur, Kerala, India; 6 Department of Biochemistry and Molecular Biology, Huck Center for Malaria Research, Pennsylvania State University, University Park, PA, USA; 7 Department of Microbiology and Immunology, Indiana University School of Medicine, Indianapolis, IN, USA; 8 Department of Immunology, Temerty Faculty of Medicine, University of Toronto, Toronto, ON, Canada; 9 Lead contact

## Abstract

T-dependent germinal center (GC) output, comprising plasma cells and memory B cells (MBCs), is crucial for clearance of *Plasmodium* infection and protection against reinfection. In this study, we examine the effect of an agonistic antibody targeting 4–1BB (CD137) during experimental malaria. We show that exogenous 4-1BB stimulation, despite delaying the effector GC response, surprisingly enhances humoral memory recall and protection from reinfection. Single-cell RNA and assay for transposase-accessible chromatin (ATAC) sequencing of MBCs from mice receiving 4-1BB stimulation reveal populations with distinct transcriptional signatures and a chromatin landscape indicative of superior recall and proliferative potential. Importantly, our results indicate that the effects of 4-1BB stimulation are dependent on interleukin (IL)-9R signaling in B cells but independent of parasite load during primary infection. Our study proposes an immunomodulatory approach to enhance the quality of the MBC pool, providing superior protection during infection and vaccination, particularly in the context of malaria.

## In brief

Calôba et al. show that exogenous 4-1BB stimulation, despite delaying the germinal center response, enhances humoral memory recall and protection against *Plasmodium* reinfection. Transcriptional and chromatin landscape analysis reveal that the protective memory B cells are extrafollicular in origin and functionally depend on B cell-intrinsic IL-9R signaling.

## Graphical Abstract

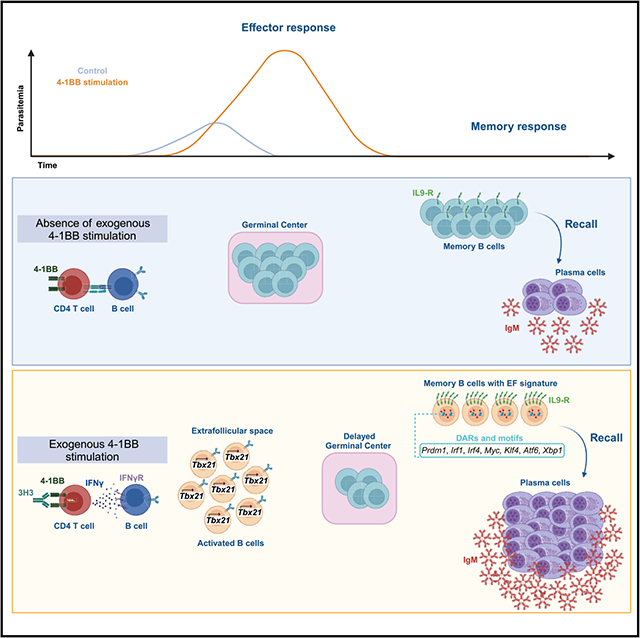

## INTRODUCTION

Despite decades of research, malaria remains a major public health threat, with an estimated 249 million cases and 608,000 deaths in 2022.^1^ The severity of the disease is especially prominent in individuals with limited or no prior exposure to the parasite, making children under the age of 5 the most impacted.^1,2^ The newly approved anti-malarial vaccines RTS,S/AS01 and R21 provide waning efficacy even after a multi-dose regimen.^3^ Clearance of the malarial parasite relies on CD4 T cell-dependent B cell activation, culminating in the production of parasite-specific antibodies during the blood stage of infection.^4,5^ Prior to or concomitant with the clearance of infection, a fraction of activated B cells differentiate into memory B cells (MBCs), which quickly become antibody-secreting plasma cells (PCs) upon reinfection.^6–12^ However, increasing evidence suggests that even after multiple exposures, naturally acquired anti-*Plasmodium* humoral immune memory affords just clinical but not sterilizing immunity, resulting in repeated infections.^13^

Leveraging numerous studies in tumor models, the expression and engagement of coinhibitory receptors such as programmed cell death protein 1 (PD-1), lymphocyte activation gene 3 (LAG-3), and cytotoxic T-lymphocyte-associated protein 4 (CTLA-4) on CD4 T cells have been identified as contributing to the inefficient anti-*Plasmodium* immune responses.^14–18^ On the other hand, exogenous activation of OX40, a costimulatory molecule belonging to the tumor necrosis factor receptor super-family (TNFRSF) expressed on T cells, improved *Plasmodium* clearance following primary infection and also rendered protection from rechallenge in a model of experimental malaria.^16,17^ These studies reveal gaps in the natural immune response to *Plasmodium* and highlight the potential of exogenous costimulatory agonists to enhance immunity.

4-1BB (CD137), another TNFRSF member predominantly expressed on T cells, has been shown to have non-redundant roles with OX40 in enhancing CD4 T cell function.^19,20^ Given that CD4 T cells play a key role in anti-*Plasmodium* immunity by providing help to B cells, we investigated the role of exogenous 4-1BB stimulation in a rodent malaria model. Here, we show that exogenous ligation of 4-1BB using a monoclonal antibody significantly delayed the effector humoral response while paradoxically enhancing humoral immune memory. Transcriptomic and chromatin accessibility analysis of MBCs from treated mice suggests that they have a distinct genetic signature that facilitates superior recall and strongly points to an extrafollicular (EF) origin. Additionally, this enhanced protection following 4-1BB activation depended on interferon-gamma (IFNγ)-induced expression of T-BET in B cells during the effector phase and, more importantly, interleukin (IL)-9:IL-9 receptor (IL-9R) signaling on MBCs. Collectively, our study reveals a novel link between two pathways programming MBCs for superior recall and identifies key targets to enhance *Plasmodium* vaccine efficacy.

## RESULTS

### Systemic 4-1BB stimulation delays effector GC response and parasite control

**Figure 1.**
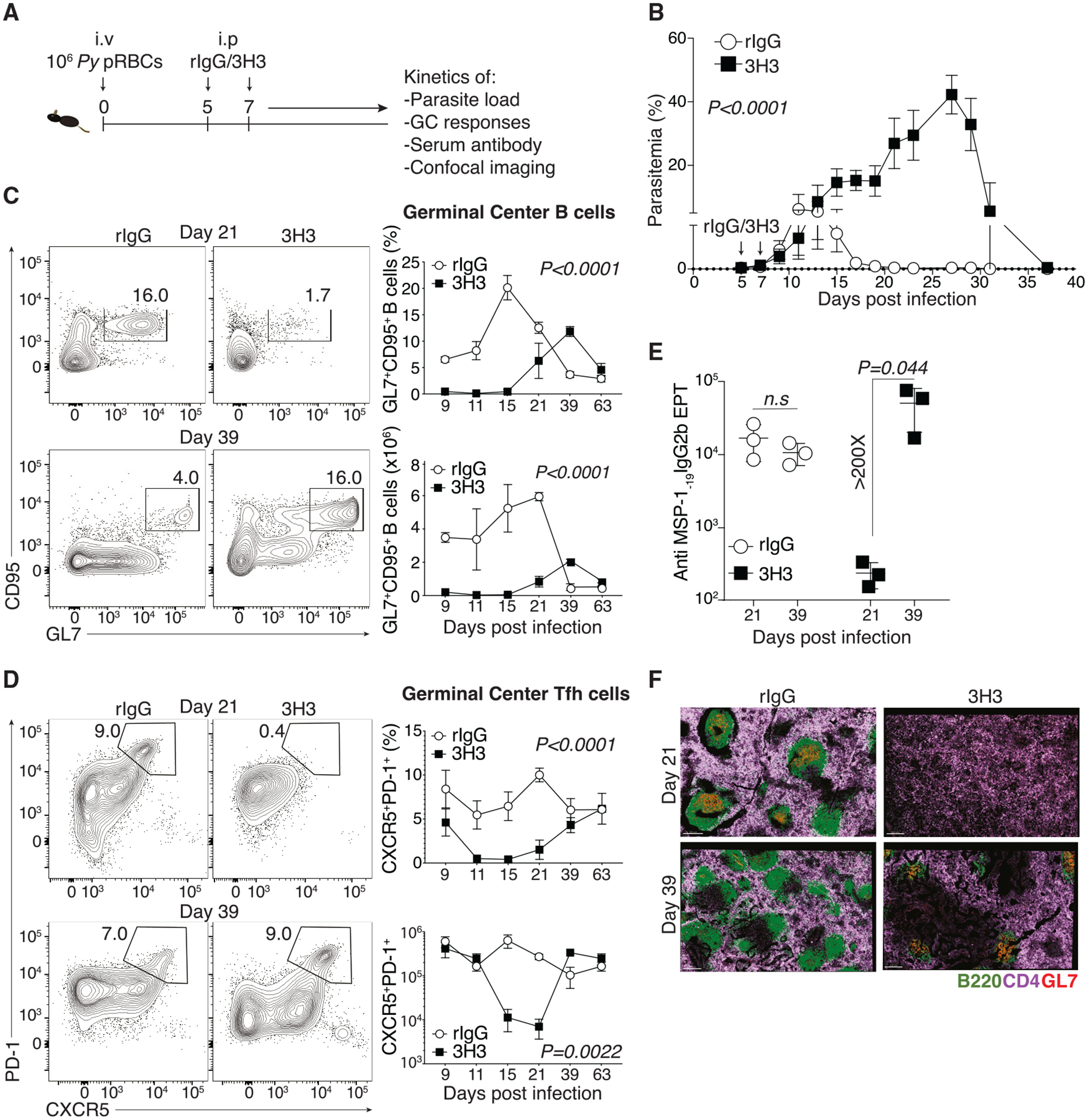
Exogenous 4-1BB stimulation derails GC responses and parasite control following *Plasmodium* infection (A) WT mice were infected with *Py* and treated with 50 μg of 3H3 or rIgG on 5 and 7 dpi. (B) Kinetics of parasite burden (% infected RBCs). Data are mean ± SEM and pooled from *n =* 2 independent experiments using *n =* 7 mice/group (C and D) Representative flow plots (left) and kinetics (right) of GC B cells (C) and GC Tfh cells (D) in spleens of rIgG- and 3H3-treated mice. (E) Summary graph of anti-MSP-1_-19_ IgG2b antibody endpoint titers (EPTs) on 21 and 39 dpi. For (C)–(E), data are mean ± SD, representative of *n =* 2 biologically independent experiments using *n =* 3 mice*/*group. (F) Confocal micrographs of rIgG- and 3H3-treated spleens on 21 and 39 dpi showing total B cells (green), CD4 T cells (purple), and GC B cells (red). Data represent at least 2 biologically independent experiments. For (F), scale bar: 200 μm. For (B)–(D), two-way ANOVA, and for (E), two-tailed Student’s t test used for statistical analysis.

Upon infection of mice with the non-lethal strain *Plasmodium yoelii* (*Py*), 4-1BB was preferentially upregulated on splenic CD4 T cells (Figure S1A). To ask whether the expression of 4-1BB may influence the outcome of infection, C57BL/6 mice (wild type [WT]) or 4-1BB^−/−^ mice were infected with *Py*, and parasite load was monitored. As shown in Figure S1B, 4-1BB^−/−^ mice showed an elevated parasite load compared to WT, suggesting a role for 4-1BB in anti-*Plasmodium* immune response. To investigate the effect of exogenous 4-1BB stimulation in the disease outcome, *Py*-infected WT mice were treated with either an agonistic antibody to 4-1BB (3H3) or isotype control (recombinant immunoglobulin G [rIgG]) on 5 and 7 days post infection (dpi) (Figure 1A), and kinetics of parasite load were monitored. As a costimulatory molecule like OX40,^21^ 4-1BB stimulation was expected to enhance the anti-*Plasmodium* humoral immune response. Surprisingly, mice treated with 3H3 exhibited a prolonged infection course and an 8-fold increase in total parasite load compared to controls, eventually resolving by 39–40 dpi (Figure 1B). Additionally, 3H3-treated mice showed a delayed onset of germinal center (GC) responses—GC B cells (Figures S1C and 1C) and GC T follicular helper (Tfh) cells (Figures S1D and 1D), peaking only around 39 dpi, suggesting that parasite clearance may be GC dependent. Not surprisingly, parasite-specific antibodies were also slow to accumulate in 3H3-treated mice (Figure 1E). Confocal imaging revealed a pronounced disruption of splenic lymphoid architecture in 3H3-treated mice, with no apparent follicular organization until 21 dpi, after which the follicles grouped into GCs as evidenced by GL7 staining (Figure 1F). Importantly, 3H3-driven loss of parasite control was reversed in mice that lacked 4-1BB in CD4 T cells (CD4^4−1BBL−/−^) (Figure S1E). These data collectively demonstrate that exogenous ligation of 4-1BB on CD4 T cells derailed GC responses and heightened parasite load following *Plasmodium* infection.

### Enhanced protection following rechallenge in mice exposed to systemic 4-1BB stimulation

**Figure 2.**
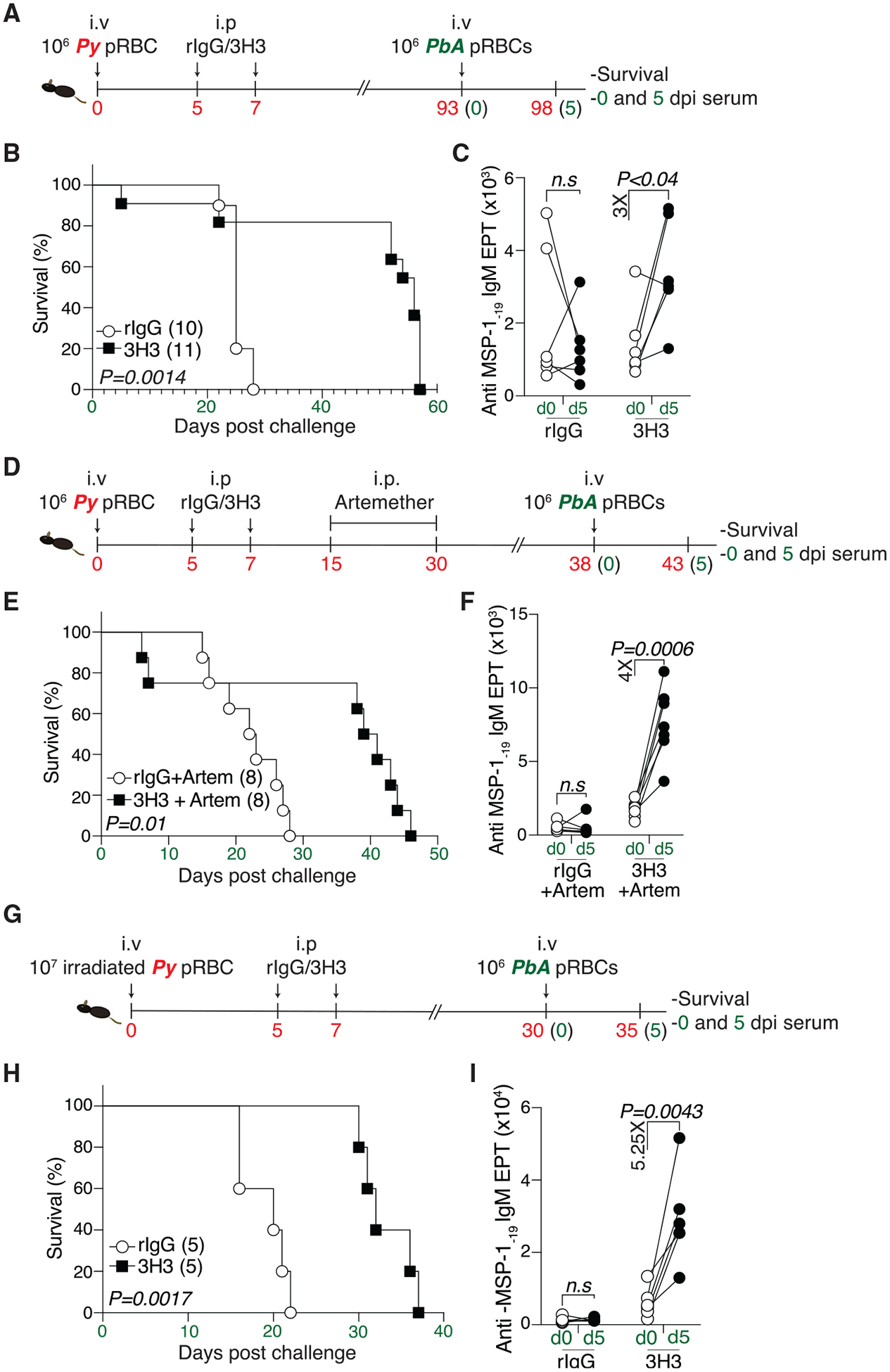
4-1BB stimulation enhances MBC recall and protection against rechallenge (A) WT mice were infected with *Py* and treated with 3H3 or isotype control. Mice were challenged with *PbA*. (B) Survival graph. Data are pooled from two independent experiments. (C) Fold change in anti-MSP-1_-19_ IgM antibody EPTs between 0 and 5 dpi. Data are pairwise comparison in each mouse pooled from *n =* 2 biologically independent experiments, using *n =* 6 (rIgG) and *n =* 5 (3H3). (D) WT mice infected with *Py* and treated with 3H3 or isotype control were treated with artemether (Artem) and challenged with *PbA*. (E) Survival graph. Data pooled from two independent experiments. (F) Fold change in anti-MSP-1_-19_ IgM antibody EPTs between 0 and 5 dpi. Data represent pairwise comparison in each mouse pooled from *n =* 2 biologically independent experiments using *n =* 7 (rIgG) and *n =* 7 (3H3). (G) WT mice vaccinated with irradiated parasites and treated with 3H3 or isotype control were rechallenged with *PbA*. (H) Survival graph. Data pooled from two independent experiments. (I) Fold change in anti-MSP-1_-19_ IgM antibody EPTs between 0 and 5 dpi. Data are pairwise comparison in each mouse pooled from *n =* 2 biologically independent experiments using *n =* 5 (rIgG) and *n =* 5 (3H3). For (B), (E), and (H), data are analyzed by Mantel-Cox test. For (C), (F), and (I), two-tailed Mann-Whitney U tests are used for statistical analysis.

Infection of mice with the invariably lethal *P. berghei ANKA* (*PbA*) strain is a stringent test for measuring MBC recall. Given that exogenous 4-1BB ligation delayed GC responses and heightened parasite burden (post *Py* infection), we hypothesized that 3H3 treatment would also impair humoral memory responses and compromise protection upon *PbA* challenge. To evaluate this, we monitored survival in convalescent *Py*-infected mice treated with either 3H3 or rIgG following *PbA* challenge on 90 days post *Py* infection. (Figure 2A). Surprisingly, 3H3-treated mice exhibited significantly longer survival than controls (Figure 2B) and accordingly exhibited a greater fold increase in anti-merozoite surface protein 1 (MSP-1_-19_)-specific antibody (IgM) titers between day 0 and day 5 post *PbA* challenge (Figure 2C), indicating an enhanced MBC recall. A rise in parasite-specific antibody titers during this short window suggests that antibodies originated from PCs differentiating from existing MBCs rather than a *de novo* GC reaction. To investigate whether elevated inflammation and/or residual antigen load from *Py* infection influenced the outcome of *PbA* challenge, we truncated the primary infection and the initial parasite load by treating mice with the antimalarial drug artemether as indicated (Figure 2D). Artemether treatment led to complete parasite clearance in both groups (Figure S2A) without affecting GC kinetics (Figures S2B and S2C), similar to non-treated mice (Figure 1). Upon *PbA* challenge, 3H3-treated mice that received chemoprophylaxis showed significantly enhanced protection (Figure 2E) and a higher fold change in anti-MSP-1 antibody titers (Figure 2F). To further test whether 3H3-mediated protection is relevant to attenuated *Plasmodium* parasite vaccination, mice were immunized with irradiated blood-stage *Py* parasites and were administered either 3H3 or rIgG on days 5 and 7 post vaccination. *PbA* challenge on days 30–35 post vaccination (Figure 2G) provided superior protection when combined with 3H3 treatment (Figure 2H), correlating with higher fold changes in serum antibody titers (Figure 2I). In summary, systemic 4-1BB ligation delays the effector GC response but significantly enhances humoral memory, independent of early antigen load.

### Enhanced humoral immune memory stems not from larger number but higher recall potential of MBCs

**Figure 3.**
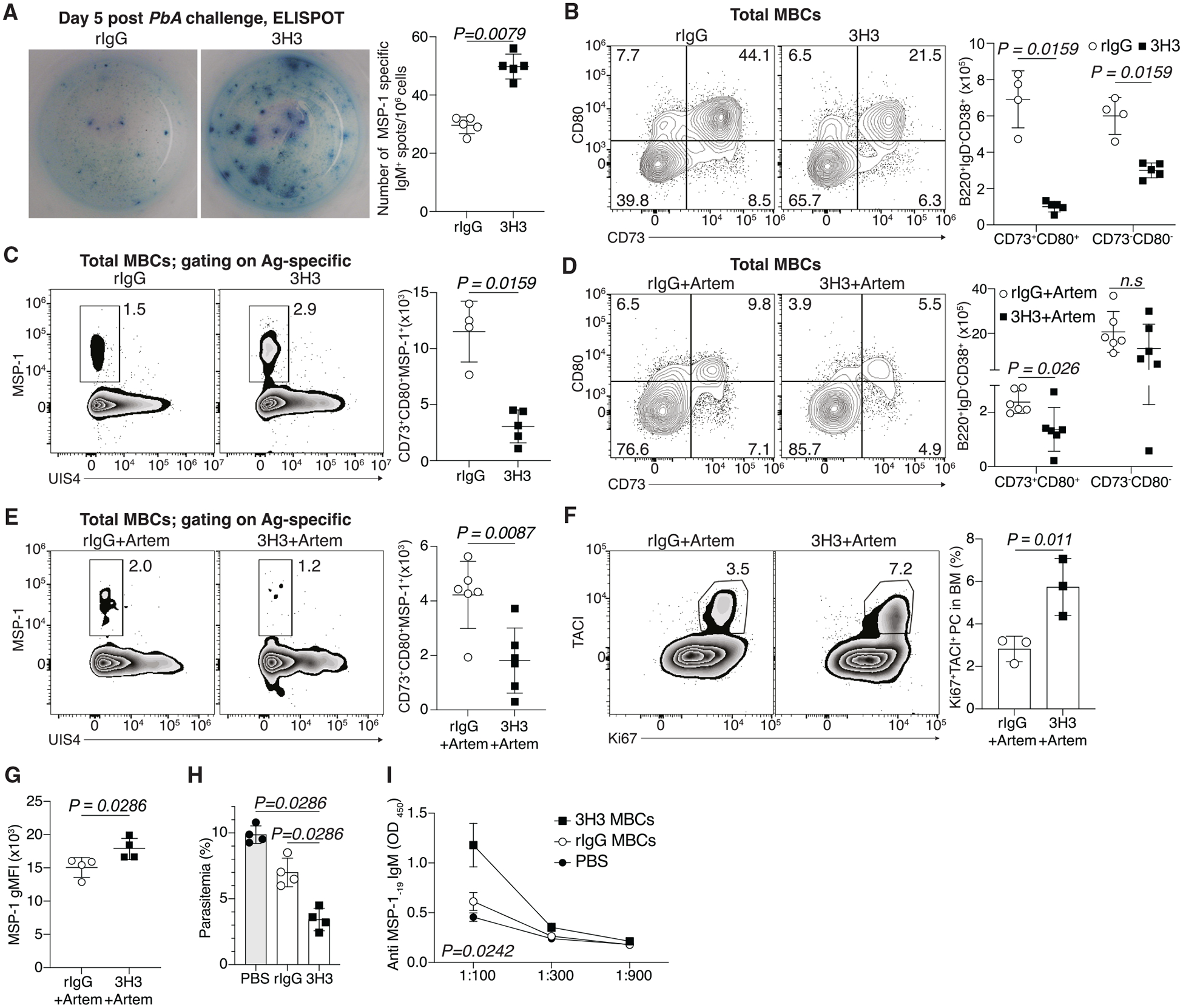
Enhanced recall potential and not MBC abundance drives protection following 4-1BB stimulation (A) Anti-MSP-1_-19_ IgM ELISPOT of BMPCs on 5 dpi post *PbA* challenge. Data are mean ± SD and represent *n =* 2 biologically independent experiments using *n =* 5 mice/group. (B–F) *Py*-infected WT mice were treated with 3H3 or isotype control. (D–F) Mice were additionally treated with Artem. Representative flow plots (left) and total numbers (right) of total (B, D) and MSP-1-specific (C, E) splenic MBCs. Data are either representative (mean ± SD) (B, C) or are pooled (mean ± SEM) from *n =* 2 biologically independent experiments using *n =* 3–4 mice/group. (F) Representative flow plots (left) and frequency (right) of proliferating BMPCs using Ki67 and TACI staining. Data are mean ± SD. (G) Geometric mean fluorescence intensity (gMFI) of MSP-1 staining in antigen-specific MBCs. Data are mean ± SD and represent *n =* 2 biologically independent experiments using *n =* 4 mice/group. (H and I) Summary graphs showing parasite burden on 12 dpi and (I) anti-MSP-1_-19_ IgM antibody titers on 4 dpi. Data are mean ± SD and represent *n =* 2 biologically independent experiments using *n =* 4 mice/group. For statistical analysis, two-tailed Mann-Whitney U test (A, B, C, D, E, G, H, and I) and Student’s t tests (F) were used.

To determine if the increased fold change in parasite-specific antibodies in 3H3-treated mice post *PbA* challenge resulted from a higher number of antibody-secreting cells, we quantified bone marrow (BM) PCs 5 days post challenge. We found a significantly greater number of MSP-1-specific PCs in 3H3-treated mice (Figure 3A). As precursors to PCs upon antigen re-exposure,^10^ we determined the frequency and number of MBCs (IgD^−^CD38^+^GL7^−^B220^+^) prior to *PbA* challenge on 90 dpi. Contrary to our expectation, 3H3-treated mice showed lower numbers of both total and antigen-specific (MSP-1 specific) (Figures S3A and S3B) MBCs compared to controls. Further characterization of the MBCs using previously described markers of functionally distinct subsets, such as CD73, CD80, and PD-L2,^22,23^ revealed a significantly lower number of total CD73^+^CD80^+^ (Figure 3B), CD73^+^PD-L2^+^, and CD80^+^PD-L2^+^ (Figures S3C and S3D) MBCs. Evaluation of MSP-1-specific MBCs also showed reduced numbers of CD73^+^PD-L2^+^ and CD80^+^PD-L2^+^ (Figures S3E and S3F) pools. MSP-1-specific MBCs within the CD73^+^CD80^+^ fraction was also lower in 3H3-treated mice (Figure 3C). The double-positive MBC fraction is known to readily differentiate into antibody-secreting cells, while the double-negative pool reseeds a secondary GC response upon antigen reencounter.^12,23^ Given that we see a rapid increase in antibody titers within 5 days of *PbA* challenge (Figure 2A), it is likely that the PCs differentiated from the existing double-positive MBCs.

To investigate the influence of chemoprophylaxis in humoral memory following 3H3 treatment, mice were infected and treated as shown in Figure 2D. Total (Figure 3D) and MSP-1-specific (Figure 3E) CD73^+^CD80^+^ MBCs were also lower in 3H3-treated mice; however, a larger frequency of BMPCs stained positive for Ki67 and transmembrane activator and CAML interactor (TACI), indicative of a high proliferative burst (Figure 3F). Taken together, these data suggest that MBCs from 3H3-treated mice are qualitatively different and are capable to differentiate into a larger number of PCs upon antigen encounter. Since artemether preserved survival and cellular phenotypes, we adopted it as a standard regimen. This approach not only accelerates convalescence (35–40 dpi vs. 85–90 dpi) but also models the chemoprophylactic approach used in malaria-endemic regions.

Confirming our prediction that MBCs from 3H3-treated mice may be functionally superior, they showed higher geometric mean fluorescent intensity (gMFI) of MSP-1 staining (Figure 3G), indicating enhanced binding affinity to MSP-1. To formally compare the functional capacity, we flow-sorted MBCs from 3H3- and rIgG-treated mice and adoptively transferred them into separate groups of B cell-deficient (μMT) recipients that received *Py* infection the following day. Mice that received MBCs from 3H3-treated donors exhibited lower parasite load compared to those receiving MBCs from rIgG-treated ones (Figure 3H), and this correlated with higher serum antibody titers (Figure 3I). Collectively, our data show that MBCs from 3H3-treated mice exhibit intrinsically superior recall potential to differentiate into a larger number of antibody-secreting PCs.

### Exogenous 4-1BB ligation drives EF MBCs

**Figure 4.**
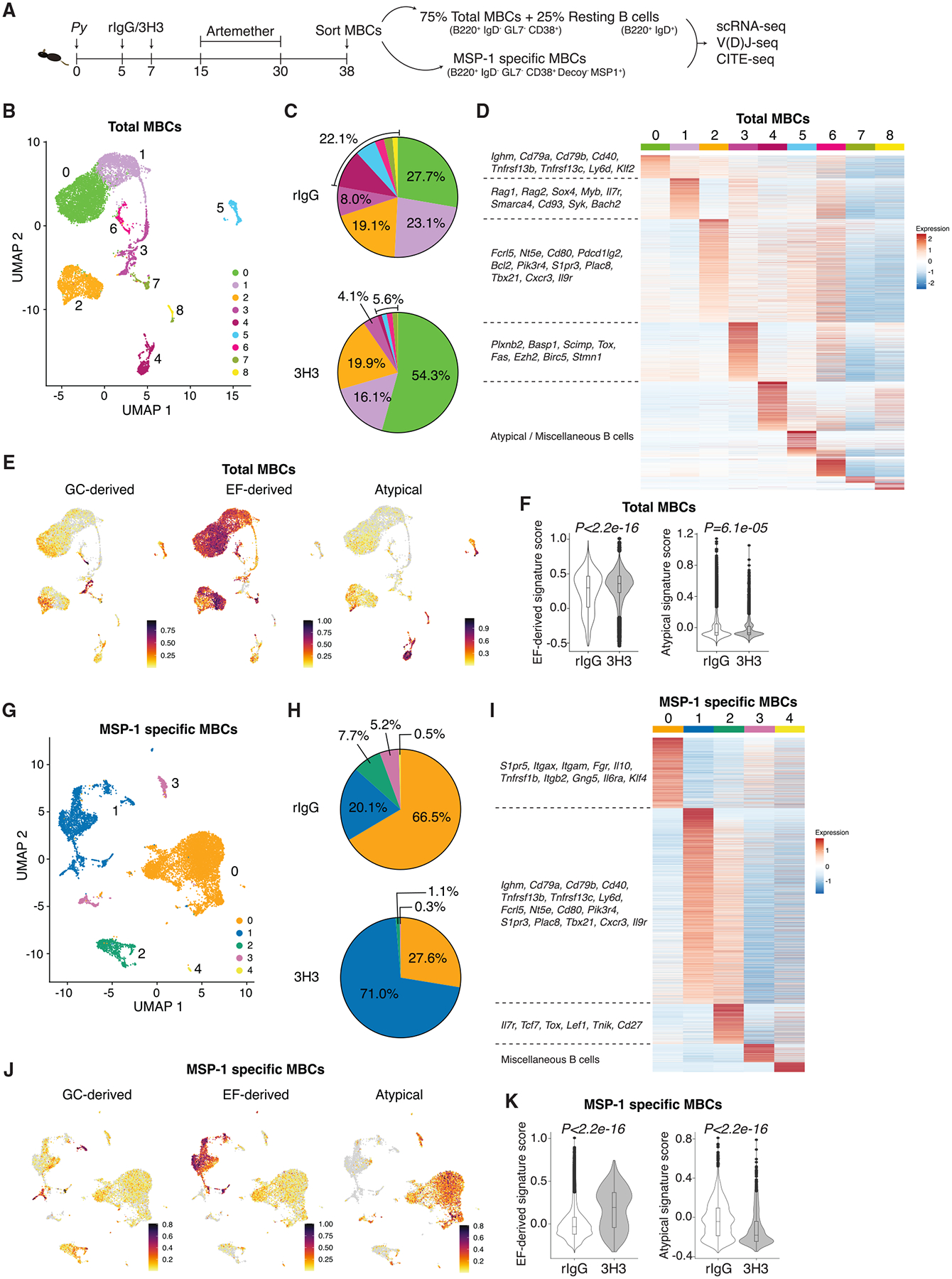
Exogenous 4-1BB ligation induces MBCs with an EF gene signature (A) WT mice were infected and treated as indicated. Total and MSP-1-specific MBCs were flow-sorted, and scRNA-seq, V(D)J sequencing, and CITE-seq were performed. (B) Uniform manifold approximation and projection (UMAP) representing identified clusters of total MBCs. (C) Pie diagram depicting the cluster frequencies in the total MBC pool derived from either group. (D) Heatmap shows DEGs with selected genes highlighted on the left. (E and F) Cells scored based on gene signatures of MBCs derived from GC or EF response or from atypical B cells and visualized per cluster (E), the prevalence of each signature is compared between treatments (F). (G) UMAP shows clusters in MSP-1-specific MBCs. (H) Frequency of clusters in MSP-1-specific MBCs from either group is shown in the pie chart. (I) Heatmap shows DEGs with selected genes highlighted on the left. (J and K) Cells scored based on gene signatures of MBCs derived from GC or EF response or atypical B cells and visualized per cluster (J) and between treatments (K). Cells pooled from 3 mice/group prior to sequencing. Wilcoxon test used for statistical analysis for data in (F) and (K).

To understand the genetic basis for the functional differences between the MBCs, single-cell RNA (scRNA), cellular indexing of trascriptomes and epitopes (CITE), and V(D)J sequencing were performed on flow-sorted total (B220^+^IgD^−^GL7^−^CD38^+^) and MSP-1-specific (B220^+^IgD^−^GL7^−^CD38^+^Decoy^−^MSP1^+^) MBCs (Figure S4A) on day 38 post *Py* infection as detailed in Figure 4A. As internal controls, resting B cells (B220^+^IgD^+^) from either group were also spiked in to represent ~25% of all cells sequenced in their respective total fraction. As shown in Figure S4B, the total MBCs grouped into 10 different clusters with the spiked-in resting B cells (IgD^+^) grouping separately (cluster 1) and representing 25%–27% of all cells sequenced (Figure S4C). To better highlight the potential differences among different MBC populations, total cells were re-clustered without the IgD^+^ population (Figure 4B) into 9 clusters. Notably, cluster 0 was significantly more represented in the 3H3 group (55%) than controls (28%) (Figure 4C). This cluster exhibited increased expression of molecules associated with B cell activation (*Cd79a*, *Cd79b*, and *Cd40*)^24–27^ and survival (*Tnfrsf13b* and *Tnfrsf13c*)^28–30^ (Figure 4D). Cluster 1, marginally overrepresented in the rIgG group (23 vs. 16%, Figure 4C), was characterized by markers associated with stemness (*Sox4*, *Myb*, *Il7r*, and *Cd93*) (Figure 4D).^31–34^ Cluster 2 was equally represented in both groups (19%–20%) (Figure 4C) and expressed genes of robust activation and recall potential such as *Fcrl5*^35^ and *Il9r*,^36^ respectively (Figure 4D). In contrast, cluster 3 was reduced in the 3H3 group (Figure 4C) and expressed genes associated with GC-derived MBCs (*Plxnb2*, *Basp1*, *Scimp*, and *Tox*)^37^ (Figure 4D). A closer look revealed that clusters 0, 1, and 2, comprising around 90% of cells in 3H3-treated mice (Figure 4C), exhibited a gene signature (Table S1) indicative of an EF origin^37^ (Figures 4E and 4F). This is consistent with our data showing a higher recall potential of MBCs from 3H3-treated mice, as EF-derived MBCs have been shown to be more proliferative and result in a larger number of PCs.^38^ Finally, clusters 4–8 that showed lower EF-derived signature comprised only 6% of MBCs in the 3H3 group compared to 22% (Figure 4C) in the rIgG group and contained mostly atypical B cells (Figures 4D–4F).

To investigate if 3H3 treatment programs antigen-specific MBCs in a similar fashion, we analyzed sequences from MSP-1-specific cells. We identified 5 clusters (Figure 4G), with cluster 0 overrepresented in the rIgG group (67%) compared to the 3H3 group (28%) (Figure 4H) and expressed genes characteristic of atypical MBCs, such as *Itgax*, *Itgam*, *Itgb2*, and *Fgr*^39,40^ (Figure 4I). Conversely, cluster 1 (71% in 3H3 vs. 20% in rIgG) (Figure 4H) was characterized by a combination of markers in clusters 0 and 2 of the total MBC pool (Figures 4D and 4I). Cluster 2 was less represented in the 3H3 group (1%) compared to the rIgG group (8%) and expressed markers associated with stemness, such as *Il7r*^41,42^ and *Lef1*^43^ (Figures 4H and 4I). Clusters 3 and 4 made up less than 6% in the rIgG group and 0.5% in the 3H3 group (Figure 4H). Much like clusters 0 and 2 in the total MBC pool (Figure 4E), cluster 1 of the MSP-1-specific pool was enriched for an EF-derived signature (Figure 4J), which was more pronounced in the 3H3 group (Figure 4K), while cluster 0 (MSP-1 specific) from the rIgG group showed the highest atypical signature (Figures 4J and 4K). These data collectively demonstrate that MBCs generated in mice following systemic 4-1BB stimulation are predominantly of EF origin, whereas those from control mice resemble atypical MBCs.

### 3H3 treatment generates MBCs with a distinct transcriptional profile supporting enhanced recall

**Figure 5.**
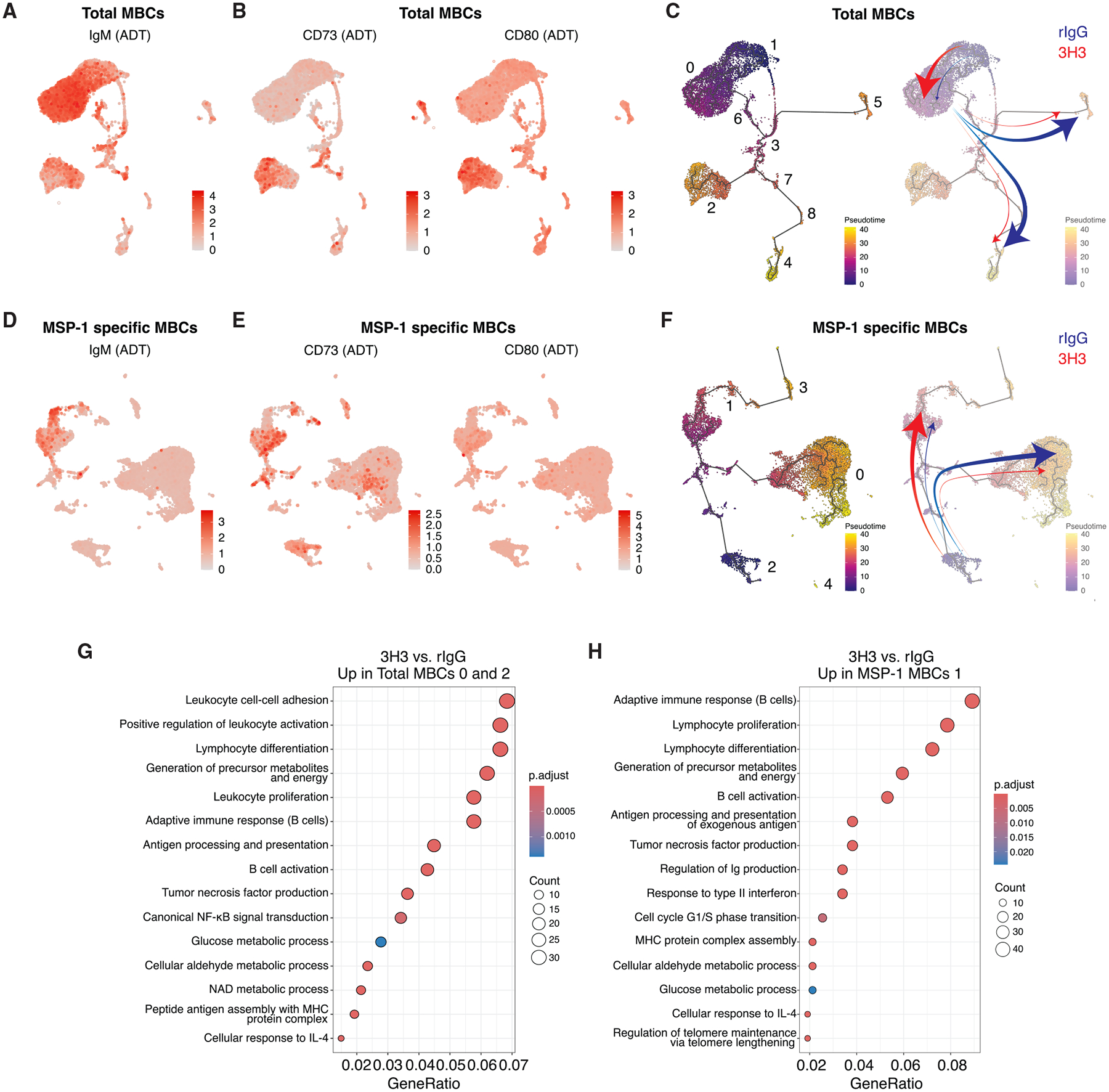
4-1BB stimulation generates highly functional MBCs (A and B) Distribution and expression of IgM (A), CD73, and CD80 (B) among the total MBCs clusters. (C) Pseudotime trajectories (left) and their representative contributions toward each cluster in total MBCs in either group (right) based on frequencies in Figure 4C. (D and E) Distribution and expression of IgM (D), CD73, and CD80 (E) among the MSP-1-specific MBC clusters. (F) Pseudotime trajectories (left) and their representative contributions in MSP-1-specific MBCs in either group (right) based on frequencies in Figure 4H. (G and H) Gene ontology (GO) analysis of upregulated genes in MBCs from 3H3-treated compared to rIgG-treated mice in clusters 0 and 2 of total MBCs combined (G) and cluster 1 of MSP-1-specific MBCs (H).

Characterization of the major MBC clusters revealed differences in the functional composition of the total and MSP-1-specific pools from rIgG- and 3H3-treated mice. Importantly, cluster 0 in the total MBC pool expressed the highest level of IgM (*Ighm*) (Figures 5A and S5A), a hallmark of highly plastic and rapidly responding MBCs following *Plasmodium* rechallenge,^38^ while cluster 2 co-expressed CD73 (*Nt5e*), CD80 (*Cd80*) (Figures 5B and S5B), and *Pdcd1lg2* (Figure S5C), markers indicative of superior recall.^23^ Utilizing pseudotime inference using cluster 1 as the origin, the IgM^+^ population (cluster 0) appeared to be in the early stage of differentiation process, while clusters 2, 4, and 5 were closer to being terminally differentiated (Figure 5C). In contrast to MBCs from rIgG-treated mice (blue arrows, Figure 5C), total MBCs from 3H3-treated mice favored the early and plastic stage^38^ (cluster 0, IgM^+^) (thick red arrow, Figure 5C) and had their differentiation skewed away from the terminal atypical trajectories (thin red arrows, Figure 5C). This will likely result in the preferential accumulation of IgM^+^ EF MBCs and reduction of atypical MBCs in the 3H3 group (Figure 4C). Characterization of MSP-1-specific pool showed that cluster 1 from 3H3-treated mice co-expressed high levels of IgM (*Ighm*) (Figures 5D and S5D), CD73 (*Nt5e*), CD80 (*Cd80*) (Figures 5E and S5E), and *Pdcd1lg2* (Figure S5F). Pseudotime analysis using cluster 2 as the origin showed that MBCs from 3H3-treated mice shifted their differentiation toward the EF trajectory (thick red arrow, Figure 5F) and away from the atypical trajectory (thin red arrow, Figure 5F), while MBCs from the rIgG group showed a higher propensity to take the atypical trajectory, away from the EF route (blue arrow, Figure 5F). The data so far showing that MBCs from 3H3-treated mice are more likely to have an EF origin are further corroborated by our V(D)J sequencing data showing significantly lower mutational frequency in their B cell receptors (BCRs) indicating a GC-independent phenotype (Figures S5G and S5H).

To explore functional differences within the EF clusters, we analyzed differentially expressed genes (DEGs) between rIgG and 3H3 in clusters 0 (IgM^+^) (Table S2A) and 2 (CD73^+^CD80^+^) (Table S2B) from the total MBC pool as well as cluster 1 from the MSP-1-specific pool (Table S2C). Notably, 122 DEGs were upregulated across all 3 clusters (Figure S5I). Approximately 88% of total MBC cluster 2 DEGs were shared with total MBC cluster 0 and/or MSP1^+^ cluster 1, with only 21 unique genes. In addition, more than half of the DEGs in total MBC cluster 0 and MSP-1-specific cluster 1 were shared, underscoring the similarities between these two clusters (Figure S5I). Given that most DEGs in total cluster 2 overlapped with the other 2 clusters (Figure S5I), we further investigated the biological processes regulated by the combined DEGs in total MBC clusters 0 and 2 (Figure 5G) and MSP-1-specific cluster 1 (Figure 5H). Notably, both total and MSP-1-specific MBCs from the 3H3 group showed increased expression of genes associated with heightened capacity for activation, proliferation, and antigen presentation as well as certain metabolic pathways, possibly to meet the energetic and biomass demands to support these processes (Figures 5G and 5H). In addition, MBCs from 3H3-treated mice also showed enhanced cellular response to IL-4, which is shown to downregulate GC-related genes and promote MBC generation and activation,^44,45^ possibly enforcing the MBCs to take the EF route. Aligning with these data, both the total (clusters 0 and 2, Figure S5J) and MSP-1-specific MBCs (cluster 1, Figure S5K) from 3H3-treated mice also downregulated genes involved in pathways leading to increased apoptosis, reduced transcription factor (TF) binding, gene silencing, and metabolism, among others. Collectively, these data confirm that exogenous ligation of 4-1BB generates MBCs with an EF origin that are, although numerically fewer, functionally superior.

### 3H3 treatment-induced protection is dependent on IFNγ and B cell-intrinsic T-BET:IL-9R axis

Studies have highlighted the role of B cell-intrinsic IFNγ signaling and T-BET expression^46–48^ for enhanced MBC recall. In agreement with a previous study,^49^ CD4 T cells from 3H3-treated mice were capable of secreting higher levels of IFNγ (Figure S6A). Additionally, B cells cultured *ex vivo* with IFNγ showed a significant increase in T-BET expression (Figure S6B), as did activated (IgD^−^) B cells from 3H3-treated mice (Figure S6C). T-BET expression in B cells during the effector phase enhances protection from rechallenge by mediating class-switching to IgG2c antibody isotype.^46^ Consistently, IgG2c levels were also markedly increased following *PbA* challenge in 3H3-treated mice (Figure S6D). To test whether 3H3-mediated superior protection is IFNγ dependent, *Py*-infected mice treated with 3H3 or rIgG and then with IFNγ-blocking antibody (XMG1.2) were *PbA* challenged, and survival was monitored (Figure S6E). As expected, 3H3-treated mice lost protection following *PbA* challenge with IFNγ blockade (Figure S6F). To test whether B cell-intrinsic T-BET expression is necessary for 3H3-mediated protection, mixed BM chimeras were generated (B^*Tbx21*−/−^) (Figure S6G) and infected with *Py*, followed by 3H3 treatment. Similar to XMG1.2 treatment, *PbA* challenge of convalescent B^*Tbx21*−/−^ mice showed significantly reduced survival compared to B^WT^ mice (Figure S6H). Together, these data show that the enhanced protection observed in mice subjected to exogenous 4-1BB ligation is dependent on the IFNγ:T-BET axis.

**Figure 6.**
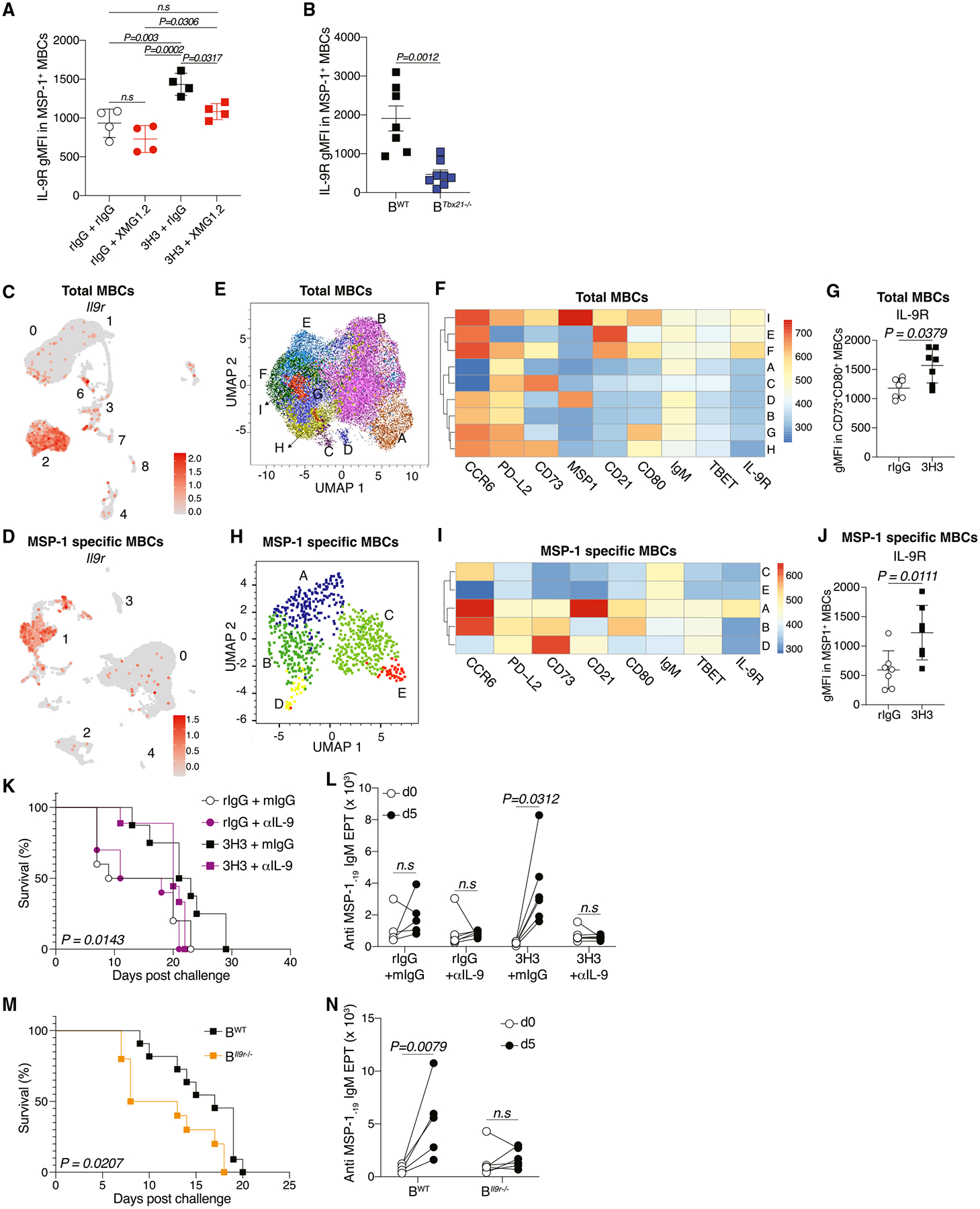
3H3-driven protection is dependent on IL-9:IL-9R signaling (A) IL-9R gMFI in MSP-1-specific MBCs on 87–89 dpi. Data are mean ± SD and represent 2 biologically independent experiments with *n =* 4 mice/group. B^*Tbx21*−/−^ or B^WT^ chimeras generated as shown in Figure S6G. (B) IL-9R gMFI in MSP-1-specific MBCs on 65 dpi. Data are mean ± SEM, pooled from 2 biologically independent experiments with *n =* 3–4 mice/group. (C and D) UMAP clustering of scRNA-seq data showing *Il9r* expression among total (C) and MSP-1-specific MBC clusters (D). (E) UMAP clustering of total MBCs on 38 dpi using FlowSOM. (F) Expression levels using FlowSOM clustering of total MBCs. (G) IL9-R gMFI in total MBCs on 38 dpi. Data are mean ± SEM, pooled from *n =* 2 biologically independent experiments using *n =* 3–4 mice/group. (H) UMAP clustering of MSP-1-specific MBCs on 38 dpi generated by FlowSOM. (I) Expression levels using FlowSOM clustering of MSP-1-specific MBCs. (J) IL-9R gMFI in MSP-1-specific MBCs on 38 dpi. Data are mean ± SEM, pooled from *n =* 2 biologically independent experiments using *n* = 3–4 mice/group. (K and L) WT mice infected with *Py* and treated with 3H3 or isotype control were treated with artemether (Artem). One day prior and after *PbA* rechallenge, mice were treated with αIL-9 neutralizing antibody or isotype control (mIgG) (Figure S7D). Survival monitored (K) and fold change in anti-MSP-1_-19_ IgM antibody EPTs between 0 and 5 dpi assessed (L). Data pooled from *n =* 2 biologically independent experiments using *n =* 6 mice/group and (L) represent pairwise comparison in each mouse. (M and N) B^WT^ and B^Il9r−/−^ chimeras generated and infected with *Py*, treated with 3H3 on 5 and 7 dpi were treated with Artem (Figure S7E). On 38 dpi, mice were *PbA* challenged. Survival monitored (M) and fold change in anti-MSP-1_-19_ IgM antibody EPTs between 0 and 5 dpi assessed (N). Data pooled from *n =* 2 biologically independent experiments using *n =* 11 mice/group (M) or represent pairwise comparison in each mouse from *n =* 2 biologically independent experiments using *n =* 5–6 mice/group (N). For (A), two-way ANOVA, for (B), (G), (J), (L), and (N), two-tailed Mann-Whitney U tests, and for (K) and (M), Mantel-Cox tests were used for statistical analysis.

IL-9R signaling on MBCs is crucial for superior recall.^36^ Accordingly, MSP-1-specific MBCs from 3H3-treated mice that received IFNγ blockade (Figure 6A), as well as Tbx21^−/−^ mice (Figure 6B), showed significantly reduced IL-9R expression, suggesting a key role for B-cell-intrinsic IFNγ-T-BET axis in its regulation. To investigate the contribution of IL-9R to higher MBC recall, we searched for distinct *Il9r*-expressing cluster(s) in our scRNA sequencing (scRNA-seq) data and identified total MBC (cluster 2) (Figure 6C) and MSP-1-specific (cluster 1) clusters (Figure 6D), which also co-expressed signature MBC markers such *as Nt5e*, *Cd80*, and *Pdcd1lg2*, among others (Figure 4D). Quantitative real-time PCR analysis on flow-sorted MBCs from 3H3-treated mice also showed significantly higher *Il9r* expression compared to controls (Figure S7A). To draw parallels among populations from our transcriptomic and flow cytometry datasets, we clustered our multi-parameter flow cytometry data (total MBCs, Figure 6E and MSP-1 specific, Figure 6H). As shown in Figures 6E and 6F, the total MBC pool clustered into 9 populations (*A*–*I*), of which *D* and *I* denoted the MSP-1-specific pool. While *E*, *F*, and *I* expressed IL-9R, *E* failed to express key functional markers such as CD73 and CD80 (Figures 6F and S7B) making it less likely to involve in a recall following antigen re-exposure. Importantly, we observed that the CD73^+^CD80^+^ MBC population from 3H3-treated mice also expressed significantly higher levels of IL-9R compared to controls (Figure 6G), suggesting that *I* may be functionally similar to *F*. Together, they may represent antigen-specific MBC pools that are swiftly recalled following antigen reencounter. Phenotypic dissimilarity of *D* and *I* (total MBCs, Figure 6F) also highlights the functional heterogeneity within a given antigen-specific pool. This was confirmed by further clustering the MSP-1-specific pool, which yielded 5 different populations, mostly differing in their expression of key functional MBC markers (Figures 6H and 6I). MSP-1-specific MBCs from 3H3-treated mice also expressed higher levels of IL-9R (Figure 6J) suggestive of their superior recall potential. Thus, total MBC cluster 2 may be identical to population *F*, and they may represent MBCs equipped to readily engage in recall. To test whether IL-9R expression is regulated by the IFNγ-T-BET axis, B cells from naive mice were activated *ex vivo* in the presence or absence of IFNγ for 3 days. Although activation of B cells alone resulted in an increase in IL-9R expression, it was further increased when IFNγ was added to the culture (Figure S7C).

To investigate whether the enhanced protection following *PbA* challenge in 3H3-treated mice is dependent on IL-9:IL-9R signaling, we blocked IL-9 in convalescent mice prior to *PbA* challenge and monitored survival (Figure S7D). As shown in Figure 6K, the enhanced protection observed in 3H3-treated mice was reversed in mice treated with IL-9-blocking antibody; rIgG-treated mice did not show any difference in survival. Supporting our observation, 3H3-treated mice subjected to IL-9 neutralization exhibited a blunted antibody recall following *PbA* challenge (Figure 6L), indicating that IL-9 signaling is important for 3H3-mediated protection. To interrogate whether B cell-intrinsic IL-9R expression is important for the enhanced protection following 3H3 treatment, competitive mixed BM chimeras were generated (B^*Il9r*−/−^) (Figure S7E) and infected with *Py*. Since no difference was observed in rIgG-treated mice following IL-9 blockade (Figure 6K), we confined our experiment to 3H3 treatment in B^*Il9r*−/−^ and B^WT^ mice. On 38 dpi, mice were challenged with *PbA*, and survival was monitored. Notably, B^*Il9r*−/−^ mice showed significantly shorter survival than B^WT^ mice following *PbA* challenge (Figure 6M) and a dampened MBC recall response (Figure 6N). These data show that the MBC recall following *PbA* challenge in 3H3-treated mice is dependent on the B cell-intrinsic IL-9R expression. Collectively, our data highlight a previously underappreciated IFNγ:T-BET:IL-9R axis in MBCs following *Plasmodium* infection, which could potentially be modulated to enhance humoral immune memory.

### Exogenous 4-1BB ligation poises MBCs for swift recall by altering their chromatin accessibility

**Figure 7.**
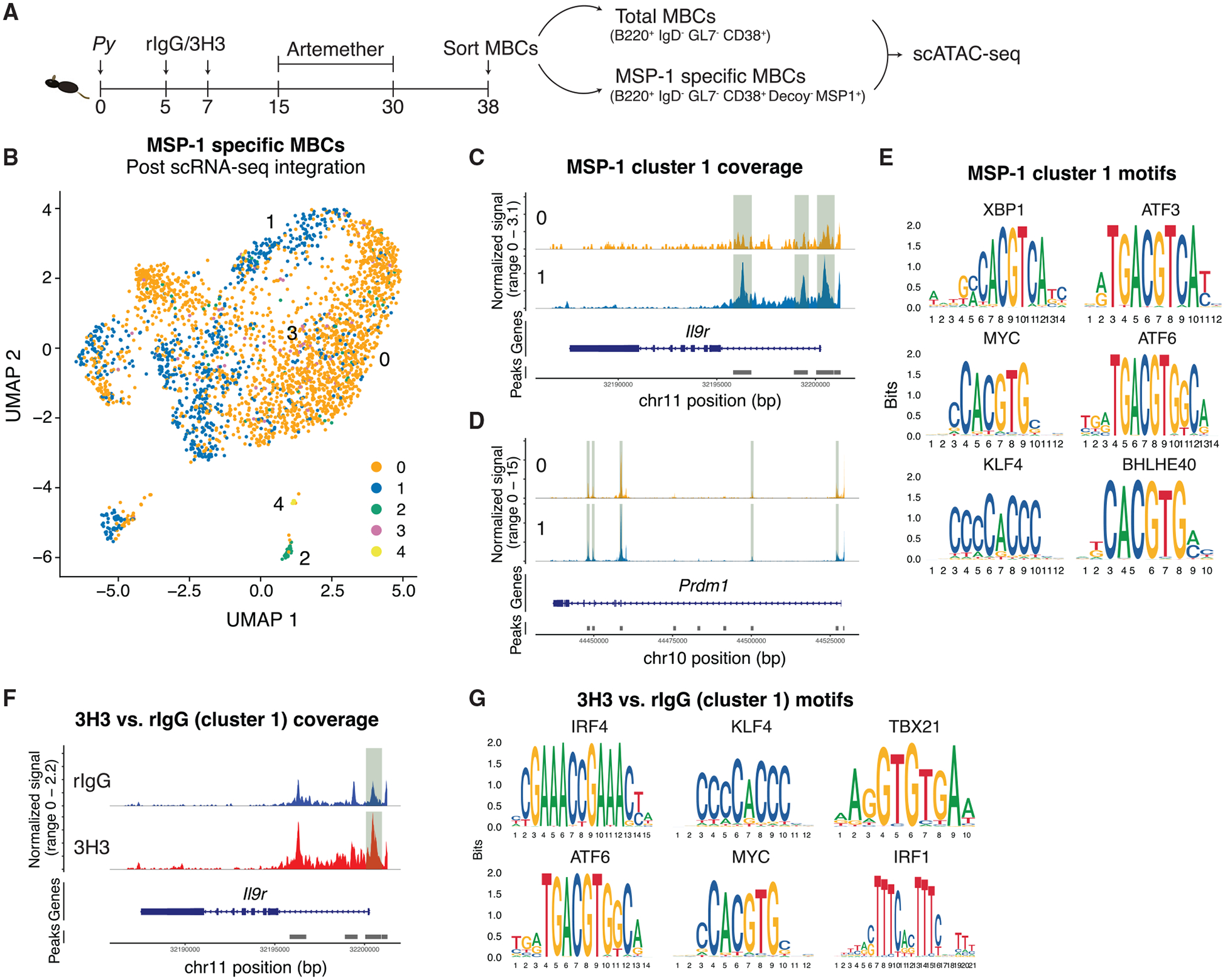
Altered chromatin landscape in MBCs following 4-1BB stimulation poises them to readily become PCs upon recall (A) WT mice infected and treated as indicated. Total and MSP-1-specific MBCs were flow-sorted and scATAC-seq performed. (B) UMAP clustering of MSP-1-specific MBCs using scATAC-seq data with transferred labels from scRNA-seq post data integration. (C and D) Comparison of *Il9r* (C) and *Prdm1* (D) accessibility between clusters 0 and 1 of MSP-1-specific MBCs. (E) Motif analysis of DARs in cluster 1 of MSP-1-specific MBCs compared to the other clusters. (F) Comparison of *Il9r* accessibility in cluster 1 between MSP-1-specific MBCs obtained from rIgG and 3H3-treated mice. (G) Motif analysis of DARs in 3H3 compared to rIgG in cluster 1 of MSP-1-specific MBCs. Sequenced nuclei pooled from 4 mice per group.

Since both total and MSP-1-specific MBCs from 3H3-treated mice downregulated genes associated with transcriptional silencing and regulation (Figures S5J and S5K), we asked whether they are imprinted with a chromatin landscape supporting superior recall. Flow-sorted total and MSP-1-specific MBCs from 3H3- or rIgG-treated mice (Figure 7A) were subjected to single-cell assay for transposase-accessible chromatin sequencing (scATAC-seq). Initial clustering based on transposase activity populated 4 different clusters in MSP-1-specific cells (Figure S8A) and 6 clusters in the total MBC pool (Figure S8B). To identify the IL-9R-expressing population in our scATAC-seq dataset and to examine the accessibility of genes that can potentiate PC differentiation, we integrated our scRNA-seq (from Figure 4) with the scATAC-seq datasets and used the transferred labels to annotate the assay for transposase-accessible chromatin clusters from MSP-1-specific (Figure 7B) and total MBCs (Figure S8C). Following integration, most cells fell in either cluster 0 or 1 (Figure 7B). Since cluster 1 in MSP-1-specific MBCs expressed the highest level of *Il9r* (scRNA-seq data, Figure 6D), we examined whether the *Il9r* gene locus in this cluster is still accessible. We observed significantly higher accessibility in the *Il9r* locus in cluster 1 compared to cluster 0 (Figure 7C), showing that MBCs in cluster 1 favor higher *Il9r* expression. In line with our prediction, cluster 1 also showed enhanced accessibility for *Prdm1* locus (Figure 7D) as well as for key TF-binding motifs (XBP1, ATF3, MYC, ATF6, KLF6, and BHLHE40) (Figure 7E) associated with enhanced PC differentiation. Importantly and similar to cluster 1 (MSP-1 specific), cluster 2 (total MBC) also showed significantly enhanced *Il9r* locus accessibility (Figure S8D) as well as markers of higher activation potential (*Fclr5*, Figure S8E), enhanced PC differentiation (*Atf6*, Figure S8F), and antiapoptotic signaling (*Bcl2*, Figure S8G). In addition, TF-binding motifs important in PC differentiation (KLF4, ATF3, XBP1, and IRF4) as well as cell proliferation (MYC) and IFNγ signaling (IRF1) were also accessible in cluster 2 from the total MBC pool (Figure S8H). We next compared the chromatin accessibility of *Il9r*-expressing MBCs between 3H3- and rIgG-treated groups. In strong agreement with our survival and phenotypic data, MBCs from 3H3-treated mice in cluster 1 not only showed continued accessibility for *Il9r* locus (Figure 7F) but were also enriched for the key TF-binding motifs associated with enhanced PC differentiation and proliferation (Figure 7G). Notably, we observed that T-BET and IRF1-binding motifs also showed higher accessibility further reinforcing our finding that the superior recall we observe in 3H3-treated mice is dependent on the IFNγ:T-BET:IL-9R axis.

## DISCUSSION

In this study, we investigated the effects of exogenous 4-1BB ligation and observed an unexpected outcome in MBC generation in an experimental mouse model of malaria. Despite the disruption of the effector GC response and a total reduction in MBC numbers, the MBC recall response was significantly enhanced, providing protection upon reinfection. While our data draw some parallels with a previous study using a viral infection model^50^ showing a derailed GC response, our findings on humoral immune memory response are unique. Single-cell transcriptomic analysis of the MBCs showed a predominant EF rather than a GC-derived signature, with IL-9R emerging as a key determinant for the enhanced recall. Using a combination of genetic, chimeric, and biochemical approaches, we identify a previously unappreciated axis comprising IFNγ and B cell-intrinsic T-BET expression during the effector phase, potentially driving the expression of IL-9R. Sequencing for transposase-accessible chromatin regions in flow-sorted MBCs from *Plasmodium-*infected mice treated with exogenous 4-1BB agonist also revealed a chromatin landscape that primed them for rapid differentiation into PCs.

*Ehrlichia muris* infection in mice results in derailed GC responses.^51,52^ Notably, the MBCs discussed in these studies were CD11c^+^ and T dependent but seldom expressed GC-related markers.^52^ The antigen-specific IgM^+^ MBCs (cluster 1) in our study are largely similar to MBCs mentioned in the aforementioned studies,^51,52^ but express FCLR5 and not CD11c. Co-expression of CD11c, CD11b, and FCLR5 is characteristic of atypical MBCs, with predominantly inhibitory functions.^53–55^ Given the lack of CD11b and CD11c expression combined with the expression of various activation markers (Figures 4 and 6) including FCLR5,^35^ we posit that the IgM^+^ MBCs from 3H3-treated mice (cluster 1) are not the inhibitory atypical MBCs.

IFNγ-driven T-BET expression is pivotal to increased protection following *Plasmodium* rechallenge.^46^ Reliance on the IFNγ:T-BET axis in 3H3-treated mice for enhanced MBC recall, along with a pronounced EF signature, is in agreement with previous findings showing that B cell-intrinsic IFNγ signaling reinforces EF B cell responses.^56^ Studies have also independently shown the role of IL-9R expression for enhanced recall.^36^ 3H3-treated but not rIgG-treated mice seem to depend on B cell-intrinsic IL-9R expression for protection, which may be explained by the far lower IL-9R expression in MBCs from control mice. Our scATAC-seq data reveal readily accessible IRF1-binding motifs in *Il9r*-expressing MBCs indicating IFNγ responsiveness.^57^ More importantly, MBCs from 3H3-treated mice have pronounced accessibility to this region along with T-BET-binding motifs. This further strengthens the possibility that IFNγ-driven T-BET may influence the expression of IL-9R in MBCs from 3H3-treated mice. We also observed lower IL-9R levels in B cells lacking T-BET or when IFNγ was blocked, which is complemented by our finding that IL-9R expression can be enhanced in B cells when subjected to IFNγ *ex vivo*. Together, these data suggest a potential role of the IFNγ:T-BET axis in IL-9R expression and raises the possibility that T-BET levels may need to reach a specific threshold to enforce IL-9R expression. While these observations are B cell centric, the source of IL-9 during memory to signal via IL-9R remains to be investigated. As such, stimulation of various TNFRSF members have been shown to promote IL-9 secretion from CD4 T cells in multiple experimental settings.^58–61^ Although our IL-9 neutralization experiments suggest the presence of an IL-9 source prevalent during the memory phase, a direct determination of the cellular identity remains to be done. IL-9 neutralization during the memory phase compromised survival and MBC recall only in 3H3-treated mice, signifying the role of IL-9:IL-9R signaling following 4-1BB stimulation. To our knowledge, our study remains the first of its kind to link the IFNγ:T-BET axis with IL-9R in MBC recall. Despite mining numerous publicly available human malaria datasets, we were unable to find a high IL-9R-expressing B cell population that could be correlated with enhanced protection upon pathogen re-exposure. These studies did not involve the use of immunomodulatory approaches that drove higher levels of T-BET in B cells, which we assume may be key to driving IL-9R. Indeed, our own *ex vivo* data with murine B cells stimulated with IFNγ resulted in the upregulation of T-BET followed by IL-9R. A fate-mapping approach capable of tracking T-BET expression would be the ideal strategy to directly ask this question, but remains beyond the scope of this manuscript.

Although many studies have focused on the effect of 4-1BB on CD8 T cells,^62,63^ systemic ligation of 4-1BB has been shown to predominantly reprogram CD4 T cells in a virus infection.^49^ It is unlikely that 4-1BB stimulation on CD8 T cells may play a significant role in our studies, as CD8 T cell depletion in 3H3-treated mice still disrupted the effector GC response and resulted in a substantial loss of parasite control, similar to control mice (*Calôba and Vijay unpublished data*). B cells are also known to express 4-1BB,^64^ but we did not explore the role of 4-1BB stimulation on B cells, as CD4-specific 4-1BB^−/−^ BM chimeras failed to respond to 3H3 treatment. These findings indicate that in rodent malaria models, the effects of exogenous 4-1BB stimulation are dependent on its expression on CD4 T cells.

Here, we provide data showing that systemic 4-1BB stimulation is capable of driving a predominantly EF B cell response generating MBCs with superior recall potential. Importantly, although the MBCs in mice that received 4-1BB stimulation are at a numerical disadvantage, a large fraction of those cells possess a distinct chromatin landscape and a transcriptional signature poising them for swift and efficient recall. Our data also bring up the argument that the GC response during the effector phase cannot always be a predictor for the ensuing memory response. We show that 4-1BB is a compelling target for host-directed immunotherapy in infections that are reliant on MBC-driven antibody response for protection. While the wide-scale use of a monoclonal antibody as an immunotherapy may be cost prohibitive, the prospect of synthesizing small-molecule activators of 4-1BB for achieving potent humoral memory immune activation and thus durable protection may be practical.

### Limitations of the study

This study demonstrates that systemic 4-1BB stimulation promotes the formation of an MBC pool with an EF signature. However, this study is limited by the absence of fate-mapping mouse models to track MBC precursors till the memory phase of infection. As a result, we were unable to precisely determine their origin or location during the effector response.

## STAR★METHODS

### EXPERIMENTAL MODEL AND STUDY PARTICIPANT DETAILS

C57BL/6 mice and *Tbx21*^−/−^ mice were purchased from The Jackson Laboratory. 4-1BB^*−/−*^ mice were originally obtained from Byoug S. Kwon, Korean National University^80^ and transferred to THW and RV labs for these studies. Mice used in the study were 6–8 weeks old and groups within each experiment were age- and sex-matched. Animals were monitored daily. *Plasmodium yoelli* (*Py*) clone 17XNL and *P. berghei* clone ANKA (*PbA*) were obtained from the Malaria Research and Reference Reagent Resource Center (MR4; American Type Culture Collection), stocks generated and mice infected as previously described.^81^ Briefly, frozen parasite stocks (200μL aliquots) were resuspended in 4mL of normal saline or PBS (1:20 dilution) and animals were infected with 1×10^6^ parasitized RBCs intravenously. All experiments and procedures were approved by the Rosalind Franklin University of Medicine and Science Institutional Animal Care and Use Committee.

### METHOD DETAILS

#### Treatments

Treatments with all biologics were done intraperitoneally in a 200μL volume (in PBS) as follows: 50 μg of anti-4-1BB antibody (3H3; BioxCell), 200μg of IFNγ blocking antibody (XMG1.2; Bioxcell), 100μg IL-9 blocking antibody (9C1; Bioxcell) or rat IgG (rIgG) or mouse IgG (mIgG) on indicated timepoints. Mice were treated with artemether (Biotechne) at 20 mg/kg, dissolved in 50μL of mineral oil at indicated timepoints.

#### Parasitemia determination

Parasitemia was measured by flow cytometry as previously described.^82,83^ Briefly, blood was collected at the indicated timepoints and were stained with the RBC marker TER-119 and nucleic acid staining dyes Hoescht-33450 and Dihydroethidium. Infected RBCs were determined by enumerating the frequency of TER119^+^ cells that stained for nucleic acid dyes.

#### Competitive mixed bone marrow chimeras

For B cell-specific competitive mixed bone marrow chimeras, C57BL/6 mice (CD45.1/.1) were lethally irradiated (950 rads) and transferred with 10×10^6^ cells containing a mixture of μMT cells (CD45.1/.2) along with *Tbx21*^−/−^ (CD45.2/.2) or *Il9r*^−/−^ (CD45.2/.2) or WT (CD45.2/.2) cells at an 8:2 ratio, respectively. For CD4 T cell-specific competitive mixed bone marrow chimeras, TCRα^−/−^ were used as recipients and transferred with 10×10^6^ cells of CD4^−/−^ cells (CD45.2/CD45.2) along with *4-*1BB^−/−^ (CD45.1/.1) or WT (CD45.1/.1) cells at an 8:2 ratio, respectively. At 6 weeks post transfer mice were checked for reconstitution (>90% donor; 80% μMT or TCRα cells and 10% KO cells) by flow cytometry and at 8 weeks post transfer, reconstituted mice were used for the experiments listed elsewhere.

#### Confocal imaging

Spleens were processed as previously described.^81^ Antibodies used for staining are B220-AF488 (clone RA3–6B2; eBioscience), CD4-AF594 (clone GK1.5; BioLegend) and GL7-AF647 (BD Pharmingen). Imaging was done using a Zeiss LSM710 confocal microscope and images were processed using IMARIS ×64 software (version 9.2.1).

#### ELISpot and ELISA

For ELISPOT, Nunc MaxiSorp white polystyrene plates were coated with 0.5 μg mL^−1^ of recombinant MSP1_19_ and blocked for at least 2 h with supplemented Roswell Park Memorial Institute (RPMI) 1640 media. Bone marrow cells from *PbA*-infected mice were serially diluted in supplemented RPMI 1640 media and plated for 20 h at 37 °C with 5% CO_2_. Plates were washed with PBS +0.05% Tween 20 and incubated overnight with horseradish peroxidase (HRP)-conjugated IgM at 4 °C. Spots were developed with 3-amino-9-ethylcarbazole.

For ELISA, Nunc MaxiSorp plates were coated with 0.5 μg mL^−1^ MSP1_19_ and blocked with 2.5% w/v BSA +5% v/v fetal bovine serum. Serum samples from naive and *Py*-infected mice were serially diluted and incubated for 18 h at 4 °C. MSP1_19_-specific antibodies were detected with HRP-conjugated IgM, IgG2b or IgG2c at 1:1000 dilution. Plates were developed using a SureBlue reserve TMB Kit (KPL), according to the manufacturer’s protocol, and absorbance was measured at an optical density of 450 nm using a Accuris ELISA plate reader. Endpoint titers were extrapolated from a sigmoidal 4PL (where x is the log concentration) standard curve for each sample. The threshold for endpoint titers was the mean plus 0.5–8 multiplied by the standard deviation recorded for naive mouse sera.

#### *Ex vivo* B cell activation

Splenic cells were enriched for B cells using the MojoSort Mouse Pan B Cell Isolation Kit (Biolegend) according to the manufacturer’s protocol. 7.5 × 10^5^ B cells were plated in triplicates and were activated with anti-IgM (10 μg/mL) and anti-CD40 (10 μg/mL). Some wells received rIFNγ (20 ng/mL).

#### Intracellular cytokine stimulation

Single cell suspension of splenocytes were prepared and 1× 10^6^ were seeded in U-bottom wells in 96-well plate. The cells were stimulated with cell activation cocktail (BioLegend) containing phorbol myristate acetate (PMA) and Ionomycin in the presence of brefeldin A (BioLegend) for 4 h at 37°C and 5% CO_2._ After incubation, cells were stained as described elsewhere in the manuscript.

#### SpyCage reagent for detecting and enriching MSP1_-19_ specific B cells

SpyCage reagents bearing 60 copies of either mScarlet (“RedCage”) or mNeonGreen (“GreenCage”) were covalently loaded with 60 copies of the immunodominant *P. yoelii* antigen MSP-1 or the liver stage antigen *Py*UIS4 as a decoy control, respectively. Antibodies specific to SpyCage were biotinylated using amine-reactive biotinylated crosslinkers and were used to enrich SpyCage-bound cells.^84^ Briefly, single cell suspensions were stained with 1.25mg of *Py*UIS4 for 10 min at room temperature followed by *Py*MSP-1 (1.25mg) staining for 30 min at 4°C. Cells were washed and incubated with 1mg of anti-aldolase biotynilated antibody for 30 min at 4°C. Stained cells were then washed and enriched with MojoSort Streptavidin nanobeads (Biolegend), according to the manufacturer’s protocol. Enriched cells were used for surface and intranuclear staining as detailed below.

#### Flow cytometry

Spleens were harvested and homogenized through wire meshes to obtain single cell suspensions. After RBCs lysis, cells were filtered and counted. Samples were blocked using Fc block (clone 2.4G2) in FACS buffer (PBS + 0.002% w/v sodium azide + 2% v/v fetal bovine serum) and cells were stained with fluorescently labeled antibodies. Transcription factors were stained using True-Nuclear Transcription Factor Buffer Set (Biolegend), according to manufacturer’s protocol. For intracellular cytokine staining Cyto-Fast Fix/Perm (Biolegend) kit was used according to manufacturer’s protocol. Samples were acquired on BD-LSR-II or Cytek Aurora and analyzed using FlowJo software (TreeStar Inc).

#### MBC transfer

Memory B cells (B220^+^IgD^−^CD38^+^GL7^−^) were sort-purified from *Py*-infected mice treated with rIgG or 3H3. 2×10^5^ cells were transferred intravenously to naive μMT mice, which were infected with 1 × 10^6^ *Py* -parasitized RBCs, the following day.

#### Single-cell RNA sequencing analysis

Single cell suspensions were stained with Live/Dead dye (Biolegend) and then divided in a ratio of 1:4 in which 1 part was used for sorting of total cells and 4 parts were used for MSP-1 specific cells. Before staining for surface markers, MSP-1 specific cells were stained and enriched with SpyCage reagents as previously described. Both total and MSP-1 cells were incubated with fluorescently labeled antibodies and Total-seq C antibodies (Biolegend) for 30 min and washed before sorting for resting B cells (B220^+^IgD^+^), total (B220^+^IgD^−^GL7^−^CD38^+^) and MSP-1 specific (B220^+^IgD^−^GL7^−^CD38^+^Decoy^−^MSP1^+^) MBCs. Post-sorted cells were run on the 10X Chromium (10X Genomics) and library preparation was performed according to the manufacturer’s protocol for Chromium Next GEM Single Cell 5′ Reagent Kit v2 with Feature Barcode technology and V(D)J amplification for mouse B cells (10X Genomics). Libraries were pooled and sequenced in the Genomics and Microbiome Core Facility at Rush University using NovaSeqX Plus (Illumina). 10X Cell Ranger Multi (v.7.1.0, 10X Genomics) was used to process gene expression, CITE-seq and V(D)J libraries in each sample individually.

Downstream analysis was performed using the Seurat package^65^ (version 5.1.0) with R (version 4.4.1). Samples were merged (rIgG and 3H3 groups in Total or MSP-1 specific pool) and the SCTransform^67^ function using the glmGamPoi^68^ package was utilized for normalization, selection of highly variable features and scaling of data. Principal component analysis (PCA) was performed and 30 dimensions were used for dimensionality reduction with Uniform Manifold Approximation and Projection (UMAP). Due to observed batch effects, the SCT datasets were integrated using the IntegrateLayers() function. Unsupervised clustering was performed using FindNeighbors() and FindClusters() function and RunUMAP() was used with reduction set to “integrated.dr”. For CITE-seq, NormalizeData() and ScaleData() were used for normalization.

Differential expression analysis was performed by PrepSCTFindMarkers() followed by FindMarkers() function, which were used to identify the markers of each cluster and differentially expressed genes (DEGs) in the 3H3 compared to rIgG group. Of the identified cluster markers, only genes with adjusted *p*-value <0.05 and average fold change >0.3 were used for the heatmaps, which show the aggregated expression of all cells within a specific cluster. Of the DEGs between 3H3 versus rIgG group, only the ones with adjusted *p*-value <0.05 were kept for gene ontology (GO) analysis. Enrichment for GO analysis was performed with the clusterProfiler package (v4.12.6)^69^ with q- and *p*-value cutoffs of 0.05 and ontology set to “Biological Processes”. To remove redundancy of enriched terms, the function simplify() was used. To generate a gene signature score for each cell in the SCT dataset, the function AddModuleScore() was used with gene lists of GC-derived, EF-derived or atypical MBCs (Table S1). Single-cell trajectories (pseudotime analysis) were constructed using the Monocle3 package.^70–74^ Cells were ordered by choosing the root cluster based on expression of genes associated with stemness and the earliest principal node was defined programmatically according to Monocle3’s vignette. RColorBrewer,^85^ ggplot2,^86^ scCustomize^87^ and viridis^88^ packages were used for visualization of the data.

#### V(D)J analysis

BCR repertoire analysis was performed using Immcantation packages.^75–79^ First, V, D and J genes were assigned using IgBLAST followed by removal of non-productive sequences and cells with multiple or without heavy chains. Then, cell IDs were matched with the gene expression data, cell type annotations were transferred and cells without gene expression data were removed. Clonal analysis was performed followed by generation of germline sequences. The V gene somatic hypermutation was calculated and used to compute the median mutation frequency of a clone. For visualization of data, the package ggplot2^86^ was used.

#### Single-cell ATAC sequencing analysis

Total and MSP-1 specific MBCs were flow-sorted as described for scRNA-seq and nuclei was isolated. Transposition, barcoding on the 10X Chromium (10X Genomics) and library construction were performed according to the manufacturer’s protocol for Chromium Next GEM Single Cell ATAC Reagents Kits v2 (10X Genomics). Libraries were pooled and sequenced in the Genomics and Microbiome Core Facility at Rush University using NovaSeqX Plus (Illumina). 10X Cell Ranger ATAC (v.2.1.0, 10X Genomics) was used to process the data.

Downstream analysis was performed using Seurat^65^ (version 5.1.0) and Signac^66^ (version 1.14.0) packages. Cells with count lower than 500 were excluded and common peak sets (for total and MSP-1 specific MBCs) were created and filtered out based on length (10000 > peak width >20). Peaks were quantified in each sample individually and the common set of peaks was passed as the feature argument. Samples were merged (rIgG and 3H3 groups in Total or MSP-1 specific pool) as Seurat objects and features that corresponded to sequences other than the standard chromosomes were removed. After gene annotation and based on the quality control metrics, cells were filtered based on the quantified peaks (100000 > quantified peaks >100), frequency of reads in peaks (20%), ratio of reads mapping in blacklist regions compared to peaks (0.08), nucleosome signal (signal <4) and transcription start site enrichment (TSS >2). Normalization, selection of top features and dimensional reduction were performed by RunTFIDF(), FindTopFeatures() and RunSVD() functions, respectively. The first LSI component was removed since it strongly correlated to sequencing depth. Clustering was performed using the FindNeighbors() function with reduction set to “lsi” followed by FindClusters() function. GeneActivity() was used to create a gene activity matrix, which was normalized and scaled by NormalizeData() and ScaleData(), respectively. FindTransferAnchors() and TransferData() functions were used to integrate scATAC-seq data with gene expression data from the scRNA-seq. Differentially accessible regions (DARs) were calculated using the FindMarkers() function using a logistic regression (LR) model. For the total DARs, it was used a threshold of adjusted *p*-value <0.05 and average log2foldchange >0.3 and for the top DARs to be used for motif analysis it was used a threshold of adjusted *p*-value <0.005 and of frequency of cells where the DAR is detected >0.2. The list of total DARs was used to calculate the closest genes to the identified peaks, while the top DARs were used to find overrepresented motifs in the accessible regions. The list of motif position frequency matrices was obtained from JASPAR2020^89^ database. was used RColorBrewer,^85^ scCustomize^87^ and ggseqlogo^90^ packages were used for visualization of the data.

#### Quantitative real-time PCR

MBCs were flow sorted from *Py*-infected mice that were treated with 3H3 or rIgG and then with artemether on time points indicated elsewhere. RNA was extracted using Trizol reagent according to the manufacturer’s protocol. 2μg of RNA was used for cDNA synthesis using MMLV reagent. cDNA was added to the 2X PCR PowerUP SYBR Green Master mix at a 1:20 ratio, along with 0.2μM of forward and reverse primers. Primer sequences used in the experiments are as follows: *Gapdh* Fwd: 5′-GAG AAC TTT GGC ATT GTG G-3’; *Gapdh* Rev: 5′-ATG CAG GGA TGA TGT TCT G-3’; *Il9r* Fwd: 5′-GGA CAG TTG GCA GTA AGT CAC C-3’; *Il9r* Rev: 5′-CCA CTC TCT CCA AGG TCC AA-3’. *Ct* value were normalized to *Gapdh* using the following equation: *ΔCt = Ct (Il9r) -Ct(Gapdh)*. Results are represented as a ratio of *Gapdh 2*^−(ΔCt)^

#### RNA data

Single cell RNA, V(D)J and CITE sequencing data are available in Gene Expression Omnibus (GEO) with the accession number GSE282525. Single cell ATAC sequencing data are available in GEO with the accession number GSE282527.

### QUANTIFICATION AND STATISTICAL ANALYSIS

Statistical analyses were performed using Prism 10 software (GraphPad). Flow cytometry data was analyzed using FlowJo v10 (BD). All *t*-tests were two-tailed and non-parametric, using a threshold of *p* < 0.05 to determine statistical significance. Specifics of each test such as values of *n*, the center, dispersion and precision measures are provided in the corresponding figure legends.

## RESOURCE AVAILABILITY

### Lead contact

Requests for additional information and resources should be directed to the lead contact, Rahul Vijay (rahul.vijay@rosalindfranklin.edu), who will facilitate their fulfillment.

### Material availability

Please contact Scott E. Lindner (sel27@psu.edu) for materials related to recombinant proteins used in this study (e.g., PyMSP1(19), PyUIS4, SpyCage scaffolds).

### Data and code availability

- scRNA-seq and scATAC-seq data are available in GEO.
- Sources of the original code are provided in STAR Methods.
- Any additional information required to reanalyze the data in this paper is available from the lead contact upon request.

## Supplementary Material

1

2

## Figures and Tables

**Table:** KEY RESOURCES TABLE

REAGENT or RESOURCE		SOURCE		IDENTIFIER
Antibodies				
*InVivo*MAb anti-mouse 4-1BB (CD137) (clone 3H3)		BioXCell		Cat #: BE0239, RRID:AB_2687721
*InVivo*MAb anti-mouse IL-9 (clone 9C1)		BioXCell		Cat #: BE0181, RRID:AB_10950648
*InVivo*MAb anti-mouse IFNγ (clone XMG1.2)		BioXCell		Cat #: BE0055, RRID:AB_1107694
*InVivo*MAb mouse IgG2a isotype control (clone C1.18.4)		BioXCell		Cat #: BE0085, RRID:AB_1107771
Rat IgG, isotype control		Sigma-Aldrich		Cat #: I4131
TotalSeq^™^-C 0450 anti-mouse IgM		BioLegend		Cat #: 406541
TotalSeq^™^-C 0571 anti-mouse IgD		BioLegend		Cat #: 405747
TotalSeq^™^-C 0225 anti-mouse CD196 (CCR6)		BioLegend		Cat #: 129829
TotalSeq^™^-C 0077 anti-mouse CD73		BioLegend		Cat #: 127237
TotalSeq^™^-C 0106 anti-mouse CD11c		BioLegend		Cat #: 117361
TotalSeq^™^-C 0914 anti-mouse CD273 (B7-DC, PD-L2)		BioLegend		Cat #: 107229
TotalSeq^™^-C 0849 anti-mouse CD80		BioLegend		Cat #: 104755
Anti-mouse T-BET, BV421-conjugated (clone 4B10)		BioLegend		Cat #: 644815, RRID:AB_2686976
Anti-mouse IgM, BV605-conjugated (clone RMM-1)		BioLegend		Cat #: 406523, RRID:AB_2563358
Anti-mouse IL-9R, PE-conjugated (clone S18011D)		BioLegend		Cat #: 158804, RRID:AB_2904303
Anti-mouse CD80, BV711-conjugated (clone 16–10A1)		BioLegend		Cat #: 104743, RRID:AB_2810338
Anti-mouse CD19, BV750-conjugated (clone 6D5)		BioLegend		Cat #: 115561, RRID:AB_2813978
Anti-mouse CD73, PE-Dazzle 594-conjugated (clone TY/11.8)		BioLegend		Cat #: 127234, RRID:AB_2800628
Anti-mouse CD11c, PE/Cy5-conjugated (clone N418)		BioLegend		Cat #: 117316, RRID:AB_493566
Anti-mouse F4/80, PE/Cy7-conjugated (clone BM8)		BioLegend		Cat #: 123114, RRID:AB_893478
Anti-mouse CD38, APC-conjugated (clone 90)		BioLegend		Cat #: 102712, RRID:AB_312932
Anti-mouse CCR6, Alexa Fluor 647-conjugated (clone 29–2L17)		BioLegend		Cat #: 129808, RRID:AB_1227497
Anti-mouse IgD, Spark NIR 685-conjugated (clone 11–26c.2a)		BioLegend		Cat #: 405750, RRID:AB_2888693
Anti-mouse CD21/CD35, APC/Fire 750-conjugated (clone 7E9)		BioLegend		Cat #: 123434, RRID:AB_2860651
Anti-mouse CD3, APC/Fire 810-conjugated (clone 17A2)		BioLegend		Cat #: 100268, RRID:AB_2876392
Anti-mouse T-BET, BV785-conjugated (clone 4B10)		BioLegend		Cat #: 644835, RRID:AB_2721566
Anti-mouse IFN-γ, PE/Cy7-conjugated (clone XMG1.2)		BioLegend		Cat #: 505826, RRID:AB_2295770
Anti-mouse B220, PerCP/Cy5.5-conjugated (clone RA3-6B2)		BioLegend		Cat #: 103235, RRID:AB_893354
Anti-mouse IgD, PE-conjugated (clone 11–26c.2a)		BioLegend		Cat #: 405705, RRID:AB_315027
Anti-mouse CD95, BV510-conjugated (clone Jo2)		BD Biosciences		Cat #: 563646, RRID:AB_2738345
Anti-mouse CD273, BV786-conjugated (clone TY25)		BD Biosciences		Cat #: 741026, RRID:AB_2740646
Anti-mouse CXCR5, PE/Cy7-conjugated (clone 2G8)		BD Biosciences		Cat #: 560617, RRID:AB_1727521
Anti-mouse CD11a, Super Bright 600-conjugated (clone M17/4)		Invitrogen		Cat #: 63-0111-82, RRID:AB_2762656
Anti-mouse PD-1, FITC-conjugated (clone RMP1–30)		Invitrogen		Cat #: 11-9981-82, RRID:AB_465467
Anti-human/mouse GL7, eFluor 450-conjugated (clone GL-7)		Invitrogen		Cat #: 48-5902-82, RRID:AB_10870775
Anti-human/mouse CD44, redFluor 710-conjugated (clone IM7)		TONBO Biosciences		Cat #: 80–0441-U100, RRID: N/A
Anti-mouse CD4, PerCP/Cy5.5-conjugated (clone GK1.5)		TONBO Biosciences		Cat #: 65–0041-U100, RRID: N/A
Purified F(ab’)2 Goat anti-mouse		BioLegend	Cat #: 157102, RRID:AB_2814087	
IgM (μ chain) (clone Poly21571)				
Ultra-LEAF^™^ Purified anti-mouse CD40 (clone FGK45)		BioLegend	Cat #: 157503, RRID:AB_2814090	
Anti-mouse CD45.1, APC/Fire 750-conjugated		BioLegend	Cat #: 110752, RRID:AB_2629805	
(clone A20)				
Anti-mouse CD45.2, FITC-conjugated (clone 104)		BioLegend	Cat #: 109806, RRID:AB_313442	
Anti-mouse TER-119, APC-conjugated (clone TER-119)		BioLegend	Cat #: 116212, RRID:AB_313712	
Anti-mouse CD137, APC-conjugated (clone 17B5)		BioLegend	Cat #: 106109, RRID:AB_2564296	
Anti-mouse CD267, PE-conjugated (clone 8F10)		BioLegend	Cat #: 133403, RRID:AB_2203542	
Anti-mouse Ki-67, BV421-conjugated (11F6)		BioLegend	Cat #: 151208, RRID:AB_2629748	
Anti-mouse B220, AF488-conjugated (clone RA3–6B2)		BioLegend	Cat #: 103225, RRID:AB_389308	
Biotin anti-mouse GL7 antigen (clone GL7)		BioLegend	Cat #: 144616, RRID:AB_2721505	
Anti-mouse CD4, AF594-conjugated (clone GK1.5)		BioLegend	Cat #: 100446, RRID:AB_2563182	
Streptavidin BV421		BioLegend	Cat #: 405226	
Peroxidase AffiniPure^™^ Goat Anti-Mouse IgM, μ chain specific		Jackson ImmunoResearch	Cat #: 115-035-075, RRID:AB_2338508	
Peroxidase AffiniPure^™^ Goat Anti-Mouse		Jackson ImmunoResearch	Cat #: 115-035-208,	
IgG, Fcγ subclass 2c specific			RRID:AB_2338516	
Chemicals, peptides, and recombinant proteins				
KPL SureBlue TMB Microwell Peroxidase Solution		SeraCare Life Sciences	Cat #: 5120-0077	
KPL TMB Stop Solution		SeraCare Life Sciences	Cat #: 5150-0021	
Tissue-Tek Optimum Cutting Temperature compound		Sakura	Cat #: IA018	
Dihydroethidium		Sigma-Aldrich	Cat #: D70008	
Hoechst 34580		Sigma-Aldrich	Cat #: 63493	
Bovine albumin serum		Sigma-Aldrich	Cat #: A7906	
N,N-Dimethylformamide		Sigma-Aldrich	Cat #: 319937	
3-Amino-9-ethylcarbazole		Sigma-Aldrich	Cat #: A6926	
MSP1_-19_ SpyCage		This paper	N/A	
*Py*UIS4 SpyCage		This paper	N/A	
Critical commercial assays				
True-Nuclear^™^ Transcription Factor Buffer Set		BioLegend	Cat #:424401	
Cyto-Fast Fix/Perm		BioLegend	Cat #:426803	
MojoSort^™^ Mouse Pan B Cell Isolation Kit II		BioLegend	Cat #:480088	
RNeasy Plus Mini kit		QIAGEN	Cat #: 28004	
Chromium Next GEM Single cell 5’ kit v2		10X Genomics	PN-1000265	
Next GEM Chip K Single Cell kit		10X Genomics	PN-1000287	
Library Construction kit		10X Genomics	PN-1000190	
Chromium Single Cell Mouse BCR amplification kit		10X Genomics	PN-1000255	
Chromium 5’ Feature Barcode kit		10X Genomics	PN-1000541	
Dual Index TT Set A		10X Genomics	PN-1000215	
Dual Index TN Set A		10X Genomics	PN-1000250	
Chromium Next GEM Single Cell ATAC Kit v2		10X Genomics	PN-1000406	
Single Index Kit N Set A		10X Genomics	PN-1000212	
Chromium Next GEM Chip H Single Cell Kit		10X Genomics	PN-1000162	
Deposited data				
scRNA-Seq		This paper	GEO: GSE282525	
scATAC-Seq		This paper	GEO: GSE282527	
Experimental models: Organisms/strains				
Model organism: Mouse: C57BL/6 (WT)		Jackson Laboratory	Stock: 00664	
Model organism: Mouse: *Tbx21 ko (C57L/6 background)*		Jackson Laboratory	Stock: 04648	
Model organism: Mouse: 4-*1BB ko (C57L/6 background)343*	Byoung S Kwon			https://pubmed.ncbi.nlm.nih.gov/12023342/
*Plasmodium yoelli*	Malaria Research and Reference Reagent Resource Center (MR4; American Type Culture Collection)			N/A
*Plasmodium berghei* ANKA	Malaria Research and Reference Reagent Resource Center (MR4; American Type Culture Collection)			N/A
Oligonucleotides				
*Gapdh* Fwd: 5’-GAG AAC TTT GGC ATT GTG G-3’	IDT			N/A
*Gapdh* Rev: 5’-ATG CAG GGA TGA TGT TCT G-3’	IDT			N/A
*Il9r* Fwd: 5’-GGA CAG TTG GCA GTA AGT CAC C-3’	IDT			N/A
*Il9r* Rev: 5’-CCA CTC TCT CCA AGG TCC AA-3’	IDT			N/A
Software and algorithms				
SpectroFlo	Cytek Biosciences			N/A
FlowJo v10.10.0	BD			https://www.flowjo.com
Prism v10.4.1	GraphPad			https://www.graphpad.com/
R v4.4.1				https://www.r-project.org
RStudio	Posit			https://posit.co/download/rstudio-desktop/
10X Cell Ranger Multi v.7.1.0	10X Genomics			https://github.com/10XGenomics/cellranger
10X Cell Ranger ATAC v.2.1.0	10X Genomics			https://github.com/10XGenomics/cellranger-atac
Seurat v5.1.0	Butler et al., 2018^65^			https://satijalab.org/seurat/r
Signac v1.14.0	Stuart et al., 2021^66^			https://stuartlab.org/signac/
SCTransform	Choudhary and Satija, 2022^67^			https://satijalab.org/seurat/articles/sctransform_vignette.html
glmGamPoi	Ahlmann-Eltze and Huber, 2021^68^			https://bioconductor.org/packages/release/bioc/html/glmGamPoi.html
clusterProfiler v4.12.6	Xu et al., 2024^69^			https://bioconductor.org/packages/release/bioc/html/clusterProfiler.html
Monocle3	Trapnell et al., 2014^70^ Levine et al., 2015^71^ Qiu et al., 2017^72^ Traag et al., 2019^73^ Cao et al., 2019^74^			https://cole-trapnell-lab.github.io/monocle3/
Immcantation	Gupta et al., 2015^75^ Gupta et al., 2017^76^ Nouri and Kleinstein, 2018^77^ Nouri and Kleinstein, 2020^78^ Hoehn et al., 2022^79^			https://immcantation.readthedocs.io/en/stable/
BioRender				https://app.biorender.com

## Highlights

- MBCs of extrafollicular origin are superior to their germinal center-derived counterparts
- Memory B cell recall depends on the IFNγ:T-BET:IL-9R signaling axis
- IL-9R expression is regulated by B cell-intrinsic T-BET
- IL-9 plays a protective role in the anti-*Plasmodium* immune response
