# Supplementary material for: Systemic 4-1BB stimulation augments extrafollicular memory B cell formation and recall responses during Plasmodium infection
Source: Cell Rep. Author manuscript; Available in PMC 2025 May 15. (PMC12079783; doi:10.1016/j.celrep.2025.115528)
Supplement: 1 [file NIHMS2076729-supplement-1.pdf]

Cell Reports, Volume *44*

## Supplemental information

# Systemic 4-1BB stimulation augments extrafollicular memory B cell formation and recall responses during *Plasmodium* infection

**Carolina Calôba, Alexandria J. Sturtz, Taylor A. Lyons, Lijo John, Akshaya Ramachandran, Allen M. Minns, Anthony M. Cannon, Justin P. Whalley, Tania H. Watts, Mark H. Kaplan, Scott E. Lindner, and Rahul Vijay**

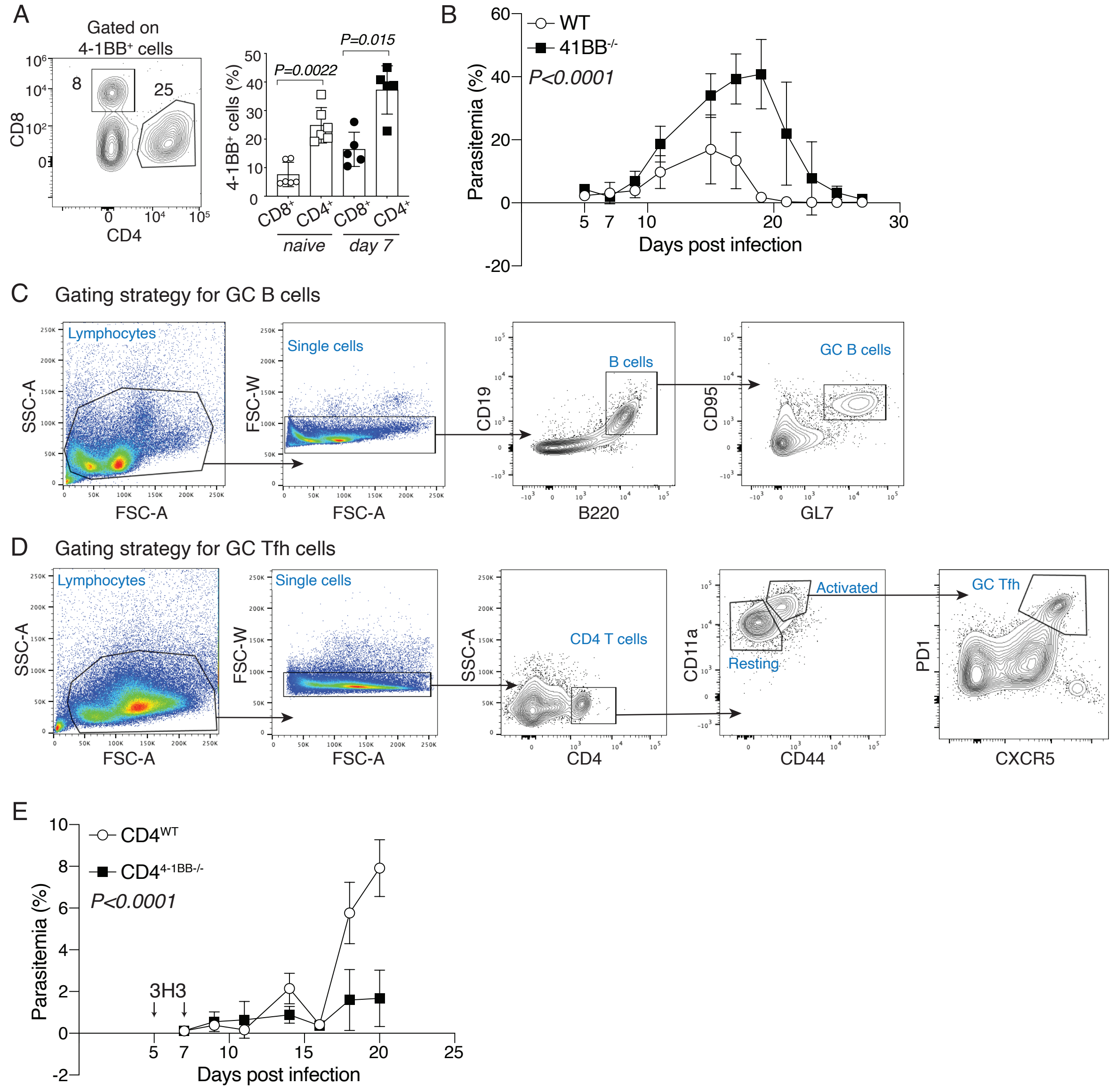


**Figure S1. 4-1BB is highly expressed in CD4 T cells during *Plasmodium* infection, Related to Figure 1.** A, Frequency of 4-1BB expressing cells in CD8 and CD4 T cell pools. Data are mean ± SEM, pooled from 2 biologically independent experiments and analyzed by two-tailed Mann-Whitney U test. B, Kinetics of parasite burden in WT and 4-1BB-/- mice. Data are mean ± SD, representative of 2 biologically independent experiments and analyzed by two-way ANOVA. C,D, Gating strategy for GC B cells (C) and Tfh cells (D). E, Kinetics of parasite burden in CD4-specific competitive mixed bone marrow chimera treated with 3H3 on 5 and 7 dpi. Data are mean ± SD, representative of 2 biologically independent experiments and analyzed by two-way ANOVA.

Supp Figure 2

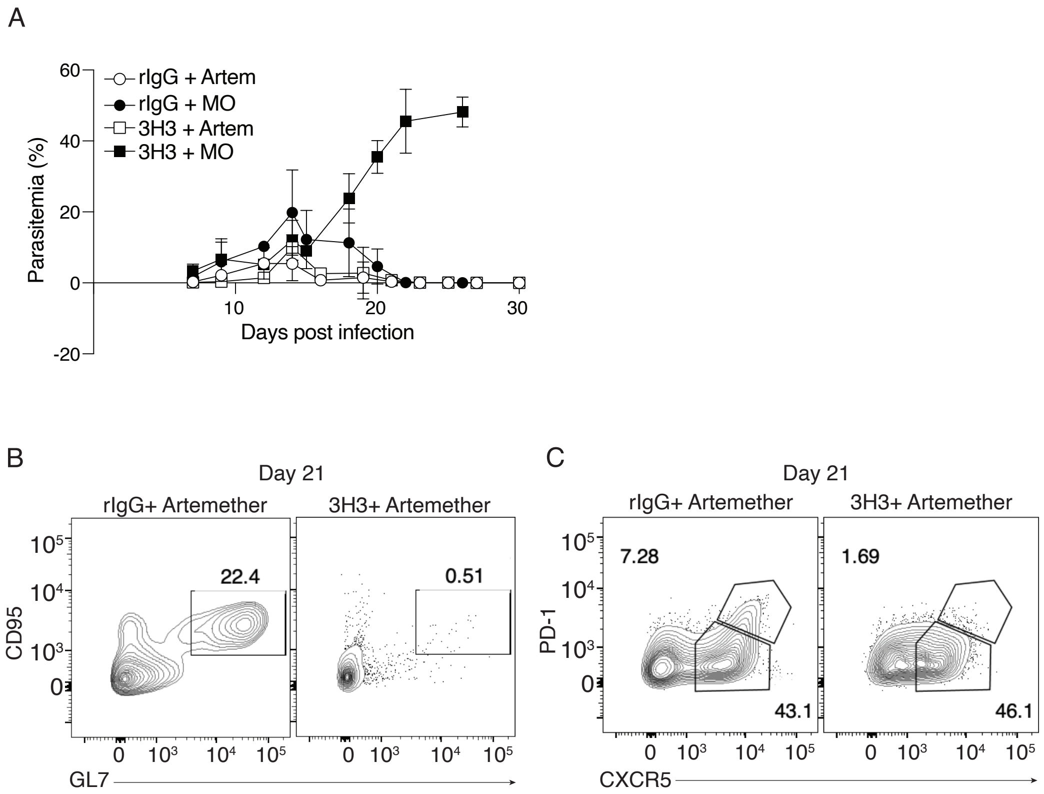


**Figure S2. 4-1BB stimulation derails GC response independently of antigen load, Related to Figure 2.** A, Kinetics of parasite burden. Data are mean ± SD, representative of at least 2 biologically independent experiments. B,C, Representative plots of GC B cells (B) and Tfh cells (C). Data are representative of at least 2 biologically independent experiments.

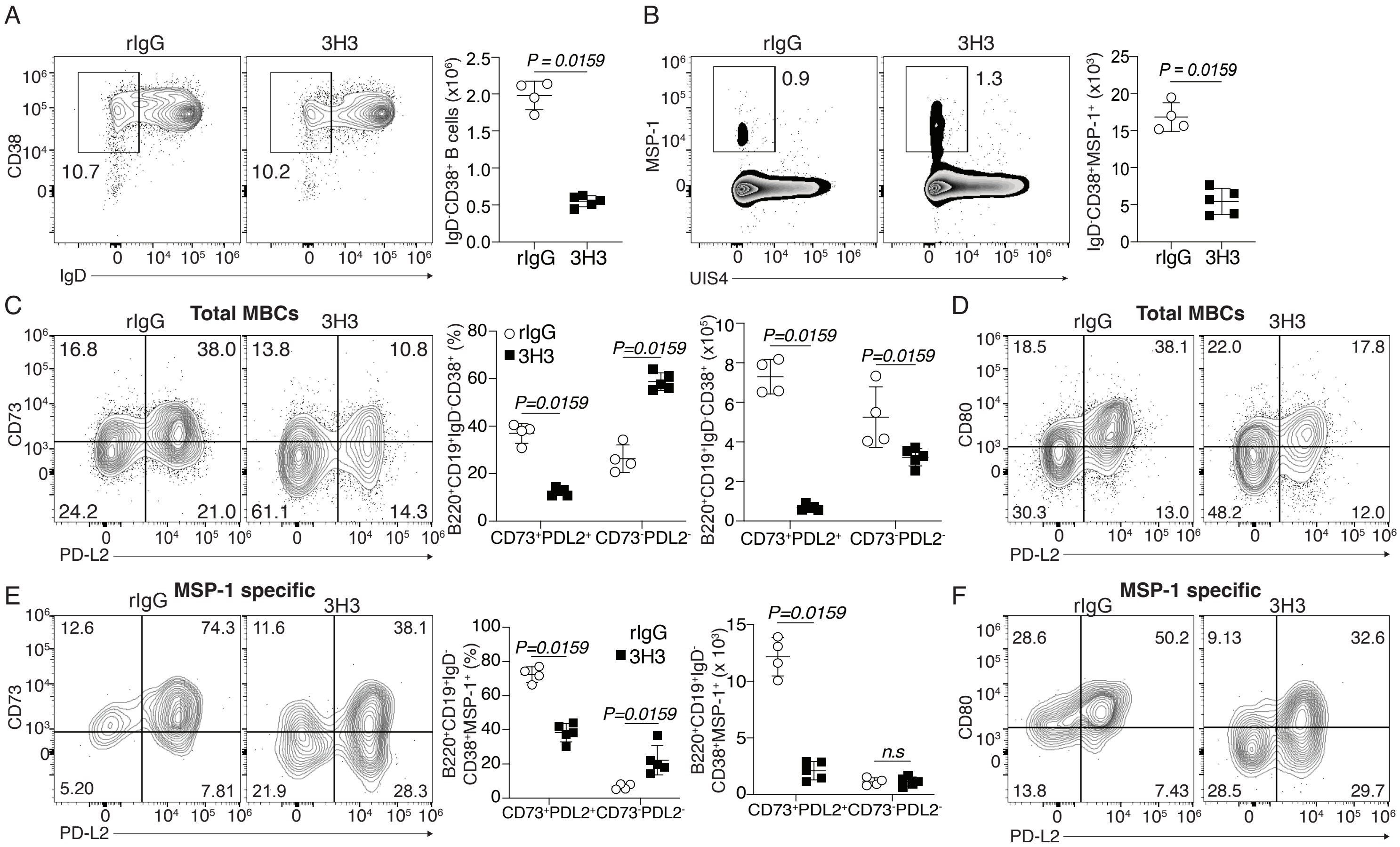


**Figure S3. Enhanced recall potential and not abundance of MBCs drives protection following 4-1BB stimulation, Related to Figure 3.** A,B, Representative plots and total numbers of total (A) and MSP-1 specific (B) MBCs. Data are mean ± SD,representative of 2 biologically independent experiments. C,D, Representative plots and total numbers of CD73+PD-L2+ (C) and CD80+ PD-L2+ (D) MBCs in the total MBC pool. Data are mean ± SD, representative of 2 biologically independent experiments. E,F, Representative plots and total numbers of CD73+PD-L2+ (E) and CD80+ PD-L2+ (F) MBCs in the MSP-1 specific pool. Data are mean ± SD, representative of 2 biologically independent experiments. All data were analyzed by two-tailed Mann-Whitney U tests.

Supp Figure 4

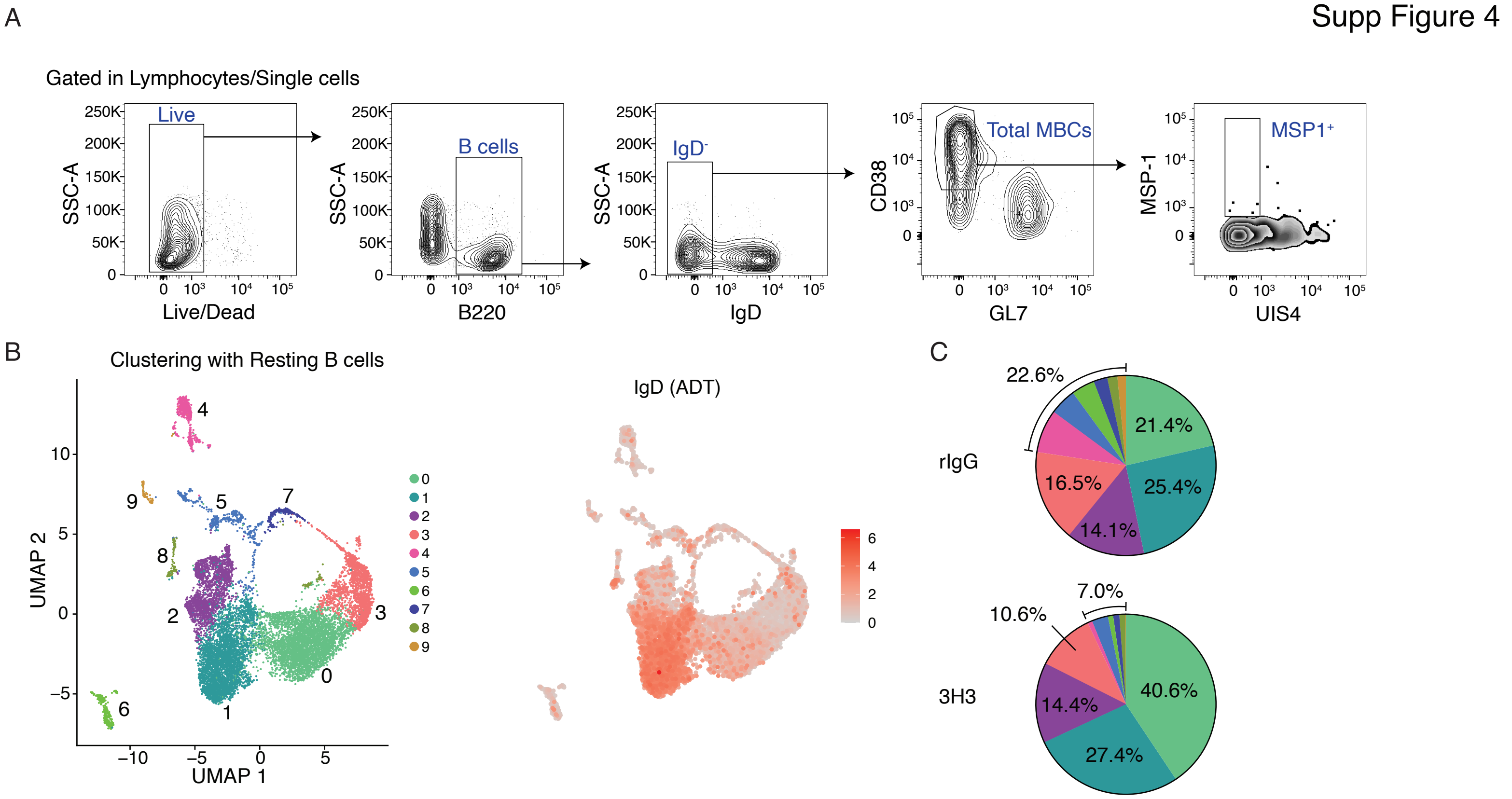


**Figure S4. Exogenous 4-1BB ligation induces MBCs with an extrafollicular gene signature, Related to Figure 4.** A, Gating strategy for sorting of resting B cells (IgD+), total and MSP-1 specific (MSP-1+) MBCs. B, UMAP clustering (left) of MBCs containing the resting B cell population (right). C, Frequency of clusters in rIgG and 3H3 cells. Sequenced cells were pooled from 3 mice per group.

Supp Figure 5

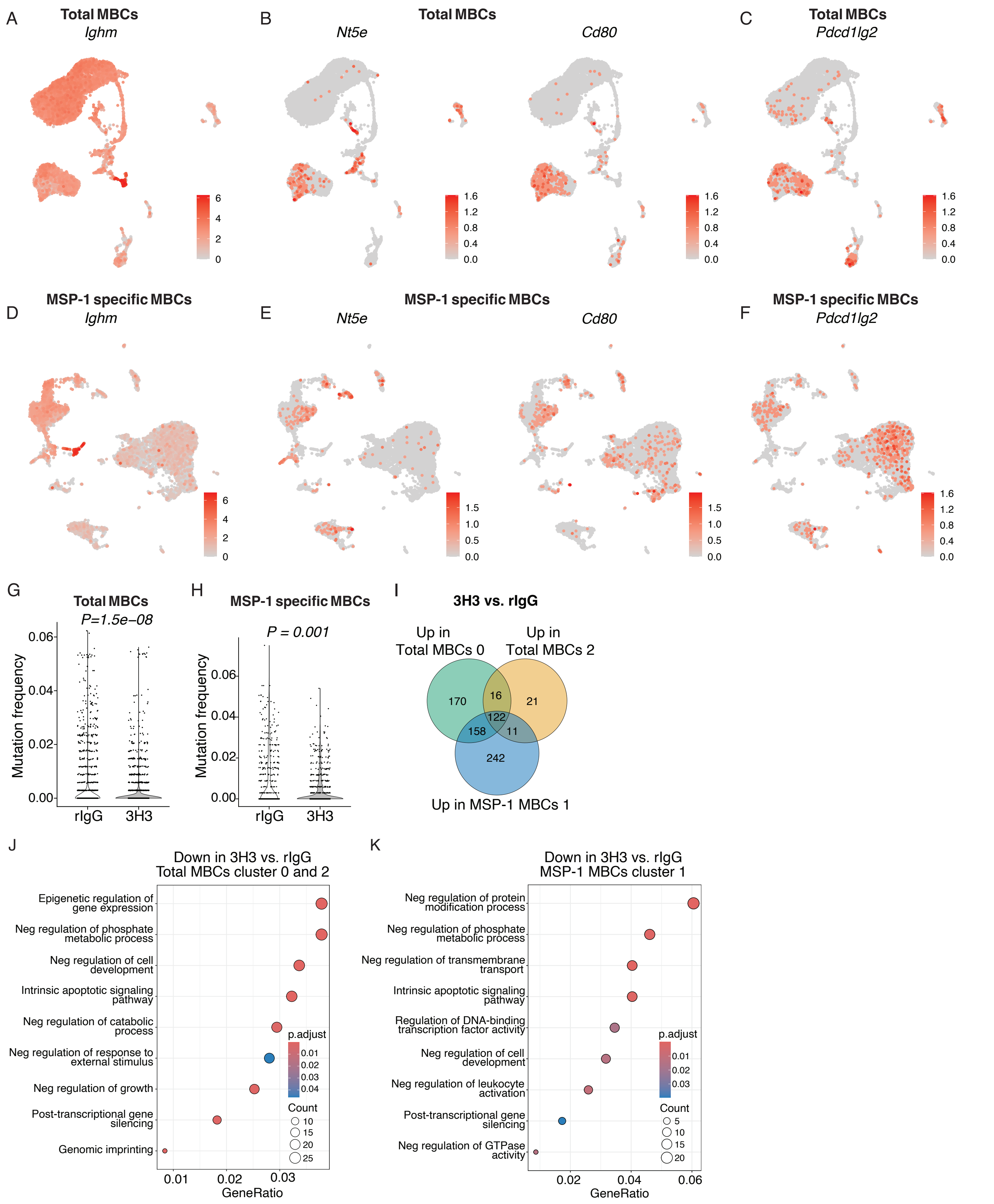


**Figure S5. 4-1BB stimulation skews MBC differentiation towards a highly functional population, Related to Figure 5.** A,B,C, Distribution of *Ighm* (A), *Nt5e* (CD73) and *Cd80* (B) and *Pdcd1lg2* (PD-L2, C) expression among the total MBCs clusters. D,E,F, Distribution of *Ighm* (D), *Nt5e* (CD73) and *Cd80* (E) and *Pdcd1lg2* (PD-L2, F) expression among the MSP-1 specific MBCs clusters. G,H, Mutation frequency of BCRs in total (G) and MSP-1 specific (H) MBCs. I, DEGs upregulated in clusters 0 and 2 of total MBCs and cluster 1 of MSP-1 specific MBCs. J,K, Gene ontology (GO) analysis of downregulated genes in MBCs from 3H3-treated mice compared to MBCs from rIgG-treated mice in clusters 0 and 2 of total MBCs combined (J) and cluster 1 of MSP-1 specific MBCs (K). Sequenced cells were pooled from 3 mice per group.

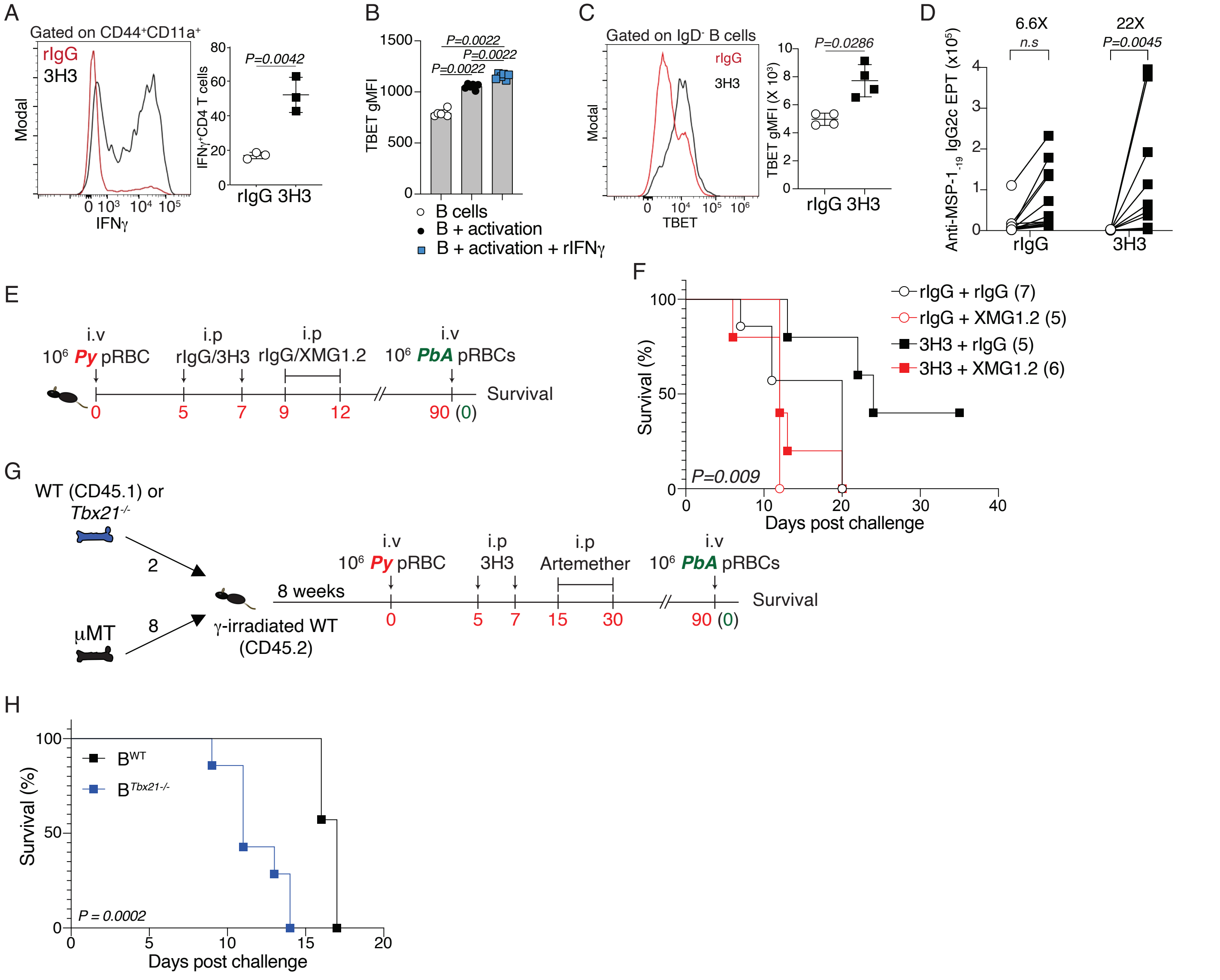


**Figure S6. 3H3-driven protection is dependent on IFNγ:T-BET signaling, Related to Figure 6.** A, Representative histogram and frequency of CD4 T cells that produce IFNγ on 15dpi. Data are mean ± SEM, pooled from at least 2 biologically independent experiments. B, gMFI of TBET in B cells that were cultured for 18h in the presence or not of IFNγ. Data are mean ± SEM, pooled from 2 biologically independent experiments with 3 replicates each. C, Representative histogram and gMFI of TBET-expressing activated B cells on 15dpi (left) and summary graph (right). Data are mean ± SD, representative of 2 biologically independent experiments. D, Fold change in anti-MSP-1-19 IgG2c serum antibody EPT between 0 and 5 dpi post *PbA* infection. Data are pairwise comparison in each mouse pooled from 2 biologically independent experiments. E,F, Experimental design (E).C57BL/6 mice were infected with *Py* and treated with 3H3 or rIgG on days 5 and 7 post infection. On days 9 to 12 post infection, mice received an IFNγ-neutralizing antibody (XMG1.2) or rIgG. During convalescence, mice were rechallenged with *PbA* and survival was monitored (F). Representative of 2 biologically independent experiments. G,H, Experimental design (G). Lethally irradiated WT mice received μMT cells together with WT or *Tbx21*$^{-/-}$ cells in a ratio of 8:2, respectively, to yield B$^{WT}$ and B$^{Tbx21-/-}$ mice. Following confirmation of reconstitution, mice were infected with *Py* 8 weeks post transfer and treated with 3H3 or rIgG on 5 and 7 dpi. During convalescence, mice were rechallenged with *PbA* and survival was monitored (H). A,B,C, and D were analyzed by two-tailed Mann-Whitney U tests. F and H were analyzed by Mantel-Cox.

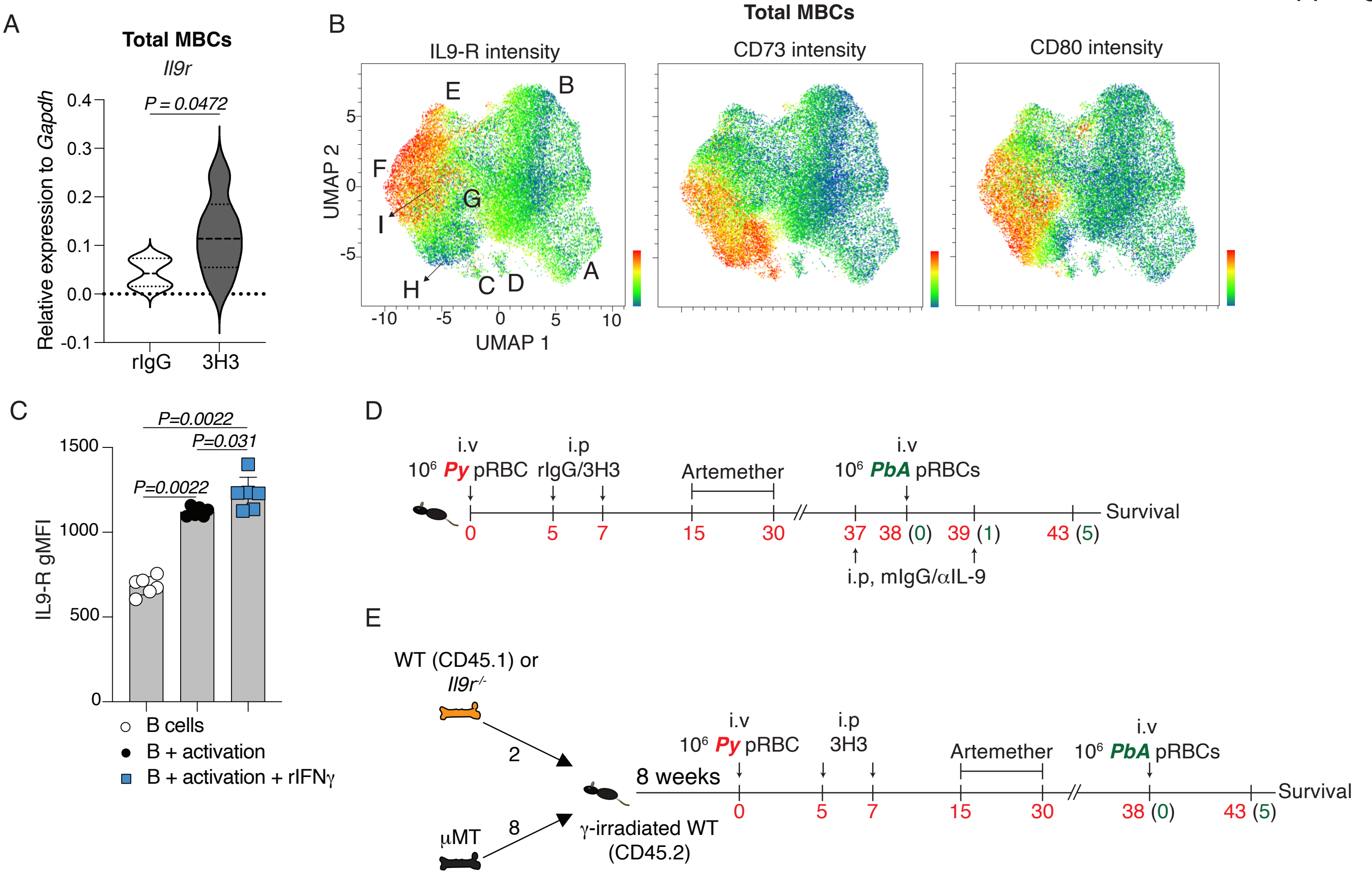


**Figure S7. 3H3-driven protection is dependent on IL-9R signaling, Related to Figure 6.** A, Real-time quantitative PCR of *Il9r* normalized with *Gapdh*. Data are mean ± SEM, pooled from at least 2 biologically independent experiments. B, Clustering of total MBCs from FlowSOM showing IL-9R, CD73 and CD80 expression among clusters. C, gMFI of IL-9R in B cells that were cultured for 3 days in the presence or not of IFNγ. Data are mean ± SEM, pooled from 2 biologically independent experiments with 3 replicates each. D, Experimental design. C57BL/6 mice were infected with *Py* and treated with 3H3 or rIgG on days 5 and 7 post infection. One day before and after *PbA* rechallenge, mice received an IL-9-neutralizing antibody or isotype control (mIgG). During convalescence, mice were rechallenged with *PbA*. E, Experimental design. During convalescence, mice were rechallenged with *PbA*. Two-tailed Student's t-test (A) and Mann-Whitney U test (C) were used for analysis.

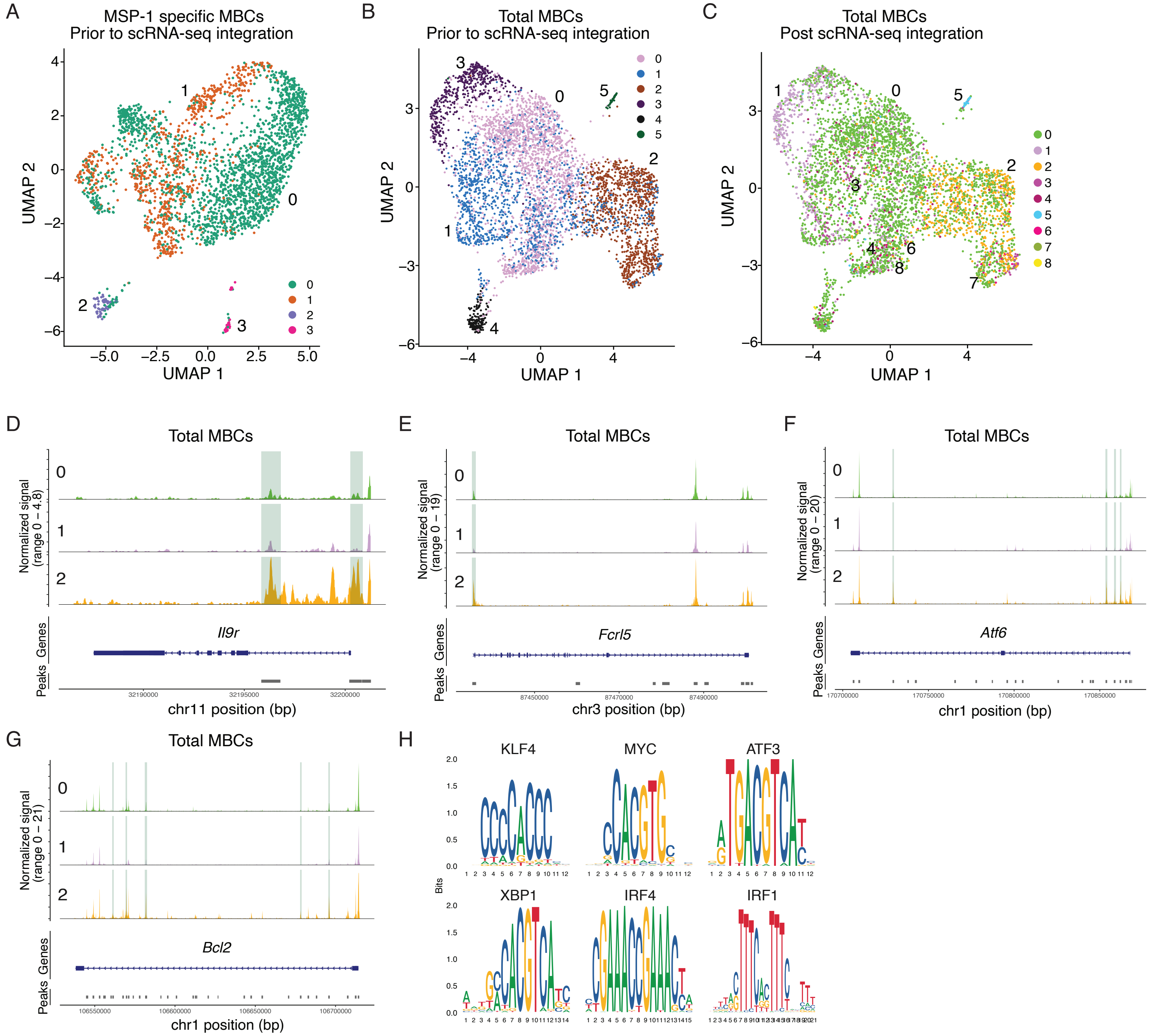


**Figure S8. Altered chromatin landscape in MBCs following 4-1BB stimulation poises them to become PCs upon recall, Related to Figure 7.** A, UMAP clustering of MSP-1 specific MBCs prior to integration with scRNA-seq data. B,C, UMAP clustering of total MBCs prior (B) and post (C) integration with scRNA-seq data. D-G, *Il9r* (D), *Fcrl5* (E), *Atf6* (F) and *Bcl2* (G) peaks in clusters 0, 1 and 2 of total MBCs. H, Motif analysis of differentially accessible regions (DARs) in cluster 2 of total MBCs. Sequenced nuclei were pooled from 4 mice per group.

| Table S1. Gene Signatures | | |
|---|---|---|
| **MBCs derived from a GC response** | **MBCs derived from an EF response** | **Atypical B cells** |
| *Nt5e* | *Ighm* | *S1pr5* |
| *Plxnb2* | *Foxp1* | *Itgax* |
| *Basp1* | *Ly6d* | *Itgam* |
| *Ighg1* | *S1pr3* | *Itgb2* |
| *Hopx* | *Plac8* | *Fgr* |
| *Scimp* | *Pik3r4* | *Itgb7* |
| *Tox* | *Tnfrsf13b* | |
| *Fcer2a* | *Gpr183* | |
| | *Tlr7* | |
| | *Tlr9* | |
